# Rbp95 binds to 25S rRNA helix H95 and cooperates with the Npa1 complex during early pre-60S particle maturation

**DOI:** 10.1093/nar/gkac724

**Published:** 2022-08-26

**Authors:** Priya Bhutada, Sébastien Favre, Mariam Jaafar, Jutta Hafner, Laura Liesinger, Stefan Unterweger, Karin Bischof, Barbara Darnhofer, Devanarayanan Siva Sankar, Gerald Rechberger, Raghida Abou Merhi, Simon Lebaron, Ruth Birner-Gruenberger, Dieter Kressler, Anthony K Henras, Brigitte Pertschy

**Affiliations:** Institute of Molecular Biosciences, University of Graz, Humboldtstrasse 50, 8010 Graz, Austria; Unit of Biochemistry, Department of Biology, University of Fribourg, Chemin du Musée 10, 1700 Fribourg, Switzerland; Molecular, Cellular and Developmental Biology Unit (MCD), Centre de Biologie Intégrative (CBI), Université de Toulouse, CNRS, UPS, 31062 Toulouse, France; Genomic Stability and Biotherapy (GSBT) Laboratory, Faculty of Sciences, Rafik Hariri Campus, Lebanese University, Beirut, Lebanon; Institute of Molecular Biosciences, University of Graz, Humboldtstrasse 50, 8010 Graz, Austria; BioTechMed-Graz, Graz, Austria; BioTechMed-Graz, Graz, Austria; Diagnostic and Research Institute of Pathology, Medical University of Graz, 8010 Graz, Austria; Institute of Molecular Biosciences, University of Graz, Humboldtstrasse 50, 8010 Graz, Austria; Institute of Molecular Biosciences, University of Graz, Humboldtstrasse 50, 8010 Graz, Austria; BioTechMed-Graz, Graz, Austria; Diagnostic and Research Institute of Pathology, Medical University of Graz, 8010 Graz, Austria; Unit of Biochemistry, Department of Biology, University of Fribourg, Chemin du Musée 10, 1700 Fribourg, Switzerland; Institute of Molecular Biosciences, University of Graz, Humboldtstrasse 50, 8010 Graz, Austria; BioTechMed-Graz, Graz, Austria; Genomic Stability and Biotherapy (GSBT) Laboratory, Faculty of Sciences, Rafik Hariri Campus, Lebanese University, Beirut, Lebanon; Molecular, Cellular and Developmental Biology Unit (MCD), Centre de Biologie Intégrative (CBI), Université de Toulouse, CNRS, UPS, 31062 Toulouse, France; BioTechMed-Graz, Graz, Austria; Diagnostic and Research Institute of Pathology, Medical University of Graz, 8010 Graz, Austria; Institute of Chemical Technologies and Analytics, Technische Universität Wien, Getreidemarkt 9/E164, 1060 Vienna, Austria; Unit of Biochemistry, Department of Biology, University of Fribourg, Chemin du Musée 10, 1700 Fribourg, Switzerland; Molecular, Cellular and Developmental Biology Unit (MCD), Centre de Biologie Intégrative (CBI), Université de Toulouse, CNRS, UPS, 31062 Toulouse, France; Institute of Molecular Biosciences, University of Graz, Humboldtstrasse 50, 8010 Graz, Austria; BioTechMed-Graz, Graz, Austria

## Abstract

Eukaryotic ribosome synthesis involves more than 200 assembly factors, which promote ribosomal RNA (rRNA) processing, modification and folding, and assembly of ribosomal proteins. The formation and maturation of the earliest pre-60S particles requires structural remodeling by the Npa1 complex, but is otherwise still poorly understood. Here, we introduce Rbp95 (Ycr016w), a constituent of early pre-60S particles, as a novel ribosome assembly factor. We show that Rbp95 is both genetically and physically linked to most Npa1 complex members and to ribosomal protein Rpl3. We demonstrate that Rbp95 is an RNA-binding protein containing two independent RNA-interacting domains. *In vivo*, Rbp95 associates with helix H95 in the 3′ region of the 25S rRNA, in close proximity to the binding sites of Npa1 and Rpl3. Additionally, Rbp95 interacts with several snoRNAs. The absence of Rbp95 results in alterations in the protein composition of early pre-60S particles. Moreover, combined mutation of Rbp95 and Npa1 complex members leads to a delay in the maturation of early pre-60S particles. We propose that Rbp95 acts together with the Npa1 complex during early pre-60S maturation, potentially by promoting pre-rRNA folding events within pre-60S particles.

## INTRODUCTION

Eukaryotic ribosomes are synthesized in a highly complex, multi-compartmental process that is initiated in the nucleolus (reviewed in (1–3)). In the budding yeast *Saccharomyces cerevisiae*, three of the four ribosomal RNA (rRNA) components present in mature ribosomes, the 18S rRNA of the 40S ribosomal subunit as well as the 5.8S and 25S rRNAs of the 60S subunit, are transcribed by RNA polymerase I as a large 35S precursor rRNA (pre-rRNA). Already co-transcriptionally, numerous ribosome assembly factors (AFs) as well as some ribosomal proteins (r-proteins) assemble with the pre-rRNA into 90S pre-ribosomal particles. These particles undergo a cascade of maturation steps mediated by more than 200 different AFs. Early processing events separate pre-40S particles and pre-60S particles, which further maturate while they move from the nucleolus to the nucleoplasm and then the cytoplasm, where the final maturation steps occur, resulting in the generation of mature small 40S and large 60S subunits.

The main types of maturation events occurring within pre-ribosomal particles are the folding, modification, and processing of the (pre-)rRNAs as well as the association of r-proteins and the binding and dissociation of AFs. Pre-rRNA processing (reviewed in (4,5)) comprises the stepwise removal of spacer elements and is additionally required to separate the maturation pathways for the large and small ribosomal subunit. It is believed that the initial pre-rRNA processing steps occur frequently co-transcriptionally in yeast (6,7). Due to this and the fact that the 18S rRNA is synthesized first as it is in the 5′ region of the common pre-rRNA precursor, most of the AFs found in 90S particles are involved in maturation of the small 40S ribosomal subunit—therefore the 90S particle is also termed SSU (small subunit) processome.

Nevertheless, 90S particles do share a few AFs with the earliest pre-60S particles, such as Rrp5, Noc1 and Noc2, suggesting that these are present during the transition from 90S to early pre-60S particles (8,9). As three-dimensional structures of the earliest pre-60S particles are still lacking, the transition from 90S to pre-60S particles and the initial steps of pre-60S particle maturation are so far only poorly understood. A protein complex formed around the scaffold protein Npa1, termed Npa1 complex, is believed to mediate key folding events in these early, still largely unstructured pre-60S particles. Notably, Npa1 contacts both the 25S rRNA 5′ domains I and II and the 3′ domain VI, thereby probably helping to physically link these domains and establish a connection that is maintained in mature ribosomes due to the interaction of r-protein L3 (Rpl3/uL3) with these rRNA elements (10).

Both 90S and early pre-60S particles undergo extensive rRNA modifications, in particular pseudouridylations and ribose methylations (reviewed in (11–13)). The majority of these modifications are introduced by small nucleolar ribonucleoprotein particles (snoRNPs), which are composed of a small RNA with complementarity to the target region and several proteins, including the enzyme introducing the modification. Whereas H/ACA snoRNPs with Cbf5 as enzymatic component are responsible for pseudouridylations, 2′-*O*-methylations are introduced by C/D box snoRNPs via the methyltransferase Nop1. Besides these snoRNPs involved in nucleotide modifications, a few snoRNPs do not catalyze any modification, but are instead involved in rRNA folding and maturation. Two such examples are the C/D box snoRNP U3 and the H/ACA snoRNP snR30, which are both required for pre-rRNA processing within 90S particles (14,15).

Here, we introduce a novel AF, Rbp95, which is a component of early pre-60S particles. *RBP95* is genetically linked to a subset of genes (*NPA1*, *NPA2*, *DBP6*, *DBP9*, *RSA3*, and *RPL3*) whose protein products are known to be involved in the assembly and maturation of early pre-60S particles. *RBP95* is hence part of the *NPA1* genetic network. Depletion of Rbp95 combined with mutation of *DBP9* leads to an accumulation of the early pre-60S specific 27SA_2_ pre-rRNA, suggesting a delay in early pre-60S particle maturation. We demonstrate that Rbp95 is an RNA-binding protein with two independent RNA-interacting domains that very specifically interacts *in vivo* with 25S rRNA helix 95 (H95) in domain VI at the 3′ end of the 25S rRNA, in physical proximity to Npa1. Moreover, we found that Rbp95 associates with several snoRNAs, particularly those base-pairing to 25S rRNA regions in proximity to helix H95. In the absence of Rbp95, maturation events taking place in vicinity to helix H95, such as the stable binding of the Ssf1-Rrp15 complex, are delayed. We propose that Rbp95 acts together with the Npa1 complex during early pre-60S maturation steps, by promoting local folding events that facilitate the subsequent stabilization of pre-60S AFs like the Ssf1-Rrp15 module.

## MATERIALS AND METHODS

### Yeast strains and genetic methods

All *S*. *cerevisiae* strains used in this study were generated by deletion and tagging at the genomic locus using established methods and they are listed in Supplementary Table S1. Yeast and *E. coli* plasmids were constructed using standard recombinant DNA techniques and they are listed in Supplementary Table S2. All DNA fragments amplified by PCR were verified by sequencing.

### Synthetic lethal screen

The synthetic lethal (SL) screen performed with the Δ*rbp95* strain was based on a combination of the *ade2*/*ade3* red/white colony-sectoring assay and counter-selection on plates containing 5-fluoroorotic acid (5-FOA, Thermo Scientific) (16–18). This approach scores for the inability to lose a plasmid carrying a *RBP95* wild-type copy, resulting in a red, non-sectoring, 5-FOA-sensitive phenotype. The SL screening strain (genotype Δ*rbp95 ade2 ade3 ura3 leu2 his3 trp1*) was transformed with the plasmid pHT4467ΔCEN-*RBP95*, carrying *ADE3* and *URA3* selection markers and a deletion in the centromeric sequence reducing its mitotic stability. The transformed strain was grown in liquid SDC medium lacking uracil (SDC-Ura) to an optical density (OD_600_) of ∼0.5 and plated on yeast extract peptone dextrose (YPD) plates at a density of about 1000 cells per plate. The plates were then UV irradiated, resulting in ∼20% survival, and incubated for 5 days at 30°C in the dark. Red colonies were streaked on YPD plates and then on 5-FOA-containing plates. Colonies with red color on YPD and inviability or strong growth phenotypes on 5-FOA were further analyzed. To confirm that the non-sectoring, 5-FOA-sensitive phenotype was due to inviability or growth defects upon loss of the pHT4467ΔCEN-*RBP95* plasmid, candidate SL strains were transformed with the *LEU2* plasmids pRS315-*RBP95* and pRS315 (empty plasmid); true SL strains should show restored sectoring and growth on 5-FOA-containing plates upon transformation with pRS315-*RBP95*, but not with pRS315. From a total of ∼100 000 screened colonies, 12 strains showed a strong synthetic enhancement (SE) of the phenotype or an SL phenotype and were thus retained for further analyses.

SL mutant #4509 was transformed with a 2μ yeast genomic tiling library bearing the *LEU2* selection marker (Dharmacon) (19). Transformants showing a red/white sectoring phenotype were re-streaked onto plates lacking leucine (SDC-Leu) and then on 5-FOA containing plates. Complementing plasmids, identified by red/white sectoring colonies on SDC-Leu and growth on 5-FOA were isolated and *RBP95* containing plasmids were identified by PCR. All other plasmids were subjected to DNA sequencing, revealing that all of them contained *NPA1*. Mutation of *NPA1* in the SL mutant #4509 was subsequently confirmed by PCR-amplification of the genomic locus and DNA sequencing.

The remaining 11 mutants were first transformed with a *NPA1*-containing *LEU2* plasmid to identify any other *NPA1* mutants. However, none of these mutants was complemented by *NPA1*, indicating that they carried mutations in other genes. Transformation with plasmids containing *NPA2*, *RPL3*, *RSA3*, *DBP6* and *DBP9* then allowed to identify the complementing genes in mutants #4512, #4510, #4627, #4511, #5009, #4514, #4506 and #4629 (see also Supplementary Table S3). The respective genes were then PCR-amplified from the genomic locus and sequenced to confirm that they indeed harbored a mutation in the SL mutants.

### Plasmid shuffle assays

*RPL3*, *DBP6* and *DBP9* shuffle strains, complemented by *URA3* plasmids bearing the respective wild-type genes were kindly provided by Jesús de la Cruz. For genetic interaction tests, *RBP95* was knocked out in these strains.

Shuffle strains with wild-type *RBP95* or *RBP95* knockout were then transformed with *LEU2* plasmids carrying different alleles of *RPL3*, *DBP6* and *DBP9*, respectively. Subsequently, the ability of the transformants to grow after loss of the *URA3* plasmid on plates containing 5-FOA was evaluated. Strains that were viable on 5-FOA plates were subsequently analyzed for their growth phenotypes on YPD.

### Tandem-affinity purification (TAP)

For TAP-purification after depletion of *RPL3*, *SSF1* and *NPA1* via the *GAL1* promoter (Supplementary Figure S8), cells grown in yeast peptone galactose (YPG) medium were used to inoculate 4 l YPG medium or 4 l YPD medium at the appropriate OD_600_ to reach an OD_600_ of ∼2 after ∼18 h of incubation.

For all other TAP purification experiments, yeast cells expressing C-terminally TAP-tagged fusion proteins were grown at 30°C in 4 l YPD to an OD_600_ of 2. TAP purifications were performed in a buffer containing 50 mM Tris–HCl (pH 7.5), 100 mM NaCl, 1.5 mM MgCl_2_, 0.1% NP-40 and 1 mM dithiothreitol (DTT). Prior to use, 1× Protease Inhibitor Mix FY (Serva) was added freshly to the buffer. Cells were lysed by mechanical disruption using glass beads. Upon clearing of the lysate by centrifugation at 4000 × g for 10 min at 4°C and a subsequent centrifugation at 30 000 × g for 30 min and 4°C, the supernatant was incubated with 300 μl IgG Sepharose™ 6 Fast Flow (GE Healthcare) at 4°C for 60 min. After incubation, beads were transferred into Mobicol columns (MoBiTec) and washed with buffer. Elution from IgG Sepharose™ beads was performed via TEV protease in the presence of 100 U RiboLock RNase inhibitor (Thermo Fisher Scientific) under rotation at room temperature (RT) for 90 min. After addition of CaCl_2_ to 2 mM concentration, TEV eluates were incubated with 300 μl Calmodulin Sepharose™ 4B (GE Healthcare) at 4°C for 60 min. After washing with 5 ml buffer containing 2 mM CaCl_2,_ pre-ribosomal particles were eluted by incubation with 600 μl buffer containing 5 mM EGTA for 30 min at RT. Samples were then either used for RNA extraction (see below), or precipitated with trichloroacetic acid (TCA), solubilized in SDS sample buffer, and then separated on NuPAGE™ 4–12% Bis–Tris gels (Invitrogen) followed by staining with NOVEX^®^ Colloidal Blue Staining Kit (Invitrogen) or western blotting. For samples to be analyzed by label-free semi-quantitative mass spectrometry (see below), elution from Calmodulin Sepharose was instead performed with 600 μl 0.8% ammonium hydroxide solution (Sigma) under rotation at RT for 20 min. The eluates were dried in a SpeedVac^®^ (Savant).

### Label-free semi-quantitative LC–MS/MS analysis of TAP eluates

Eluates from TAP purifications were dissolved in 25% 2,2,2-trifluoroethanol (TFE) in 50 mM Tris–HCl (pH 8.5), reduced with 10 mM Tris(2-carboxyethyl)phosphine (TCEP) and alkylated with 40 mM chloroacetamide by shaking at 550 rpm at 95°C for 10 min. After dilution to <10% TFE with 50 mM ammonium bicarbonate, proteins were digested by adding 1 μg of Promega modified trypsin and shaking overnight at 550 rpm at 37°C. The resulting peptide solution was acidified by adding 5% formic acid to a final concentration of 0.1% and analyzed by nano-HPLC coupled to either an Orbitrap Velos Pro (Thermo Fisher Scientific) or a maXis II ETD (Bruker) mass spectrometer.

For Orbitrap measurements a Dionex Ultimate 3000 was equipped with a C18 (5 μm, 100 Å, 5 × 0.3 mm) enrichment column and an Acclaim PepMap RSLC C18 nanocolumn (2 μm, 100 Å, 500 × 0.075 mm) (all Thermo Fisher Scientific).

Samples were concentrated on the enrichment column for 6 min using 0.1% formic acid as isocratic solvent at 5 μl/min flow rate. The column was then switched in the nanoflow circuit, and the sample was loaded on the nanocolumn, at a flow rate of 250 nl/min at 60°C and separated using the following gradient: solvent A: water, 0.1% formic acid; solvent B: acetonitrile, 0.1% formic acid; 0–6 min: 4% B; 6–264 min: 4–25% B; 264–274 min: 25–95% B, 274–289 min: 95% B; 289–304 min: 4% B. The sample was ionized in the nanospray source equipped with stainless steel emitters (Thermo Fisher Scientific) and analyzed in the Orbitrap Velos Pro mass spectrometer in positive ion mode by alternating full scan MS (*m*/*z* 300 to 2000, 60 000 resolution) in the ICR cell and MS/MS by CID of the 10 most intense peaks in the ion trap with dynamic exclusion enabled.

For maXis II ETD measurements peptide solutions were acidified to a final concentration of 1% Trifluoroacetic acid and desalted before measurement using in-house made stage-tips with SDB-RPS (styrene divinylbenzene-reversed phase sulfonate) as material. The Dionex Ultimate 3000 was equipped with an Aurora Series Emitter nanocolumn with CSI fitting (C18, 1.6 μm, 120 Å, 250 × 0.075 mm) (IonOpticks, Melbourne, Australia). Separation was carried out at 50°C at a flow rate of 300 nl/min using the following gradient (same solvent A and B as described above): 0–18 min: 2% B; 18–100 min: 2–25% B; 100–107 min: 25–35% B, 107–108 min: 35–95% B; 108–118 min: 95% B, 118–118 min: 95–2% B; 118–133 min: 2% B. The Bruker maXis II ETD mass spectrometer was operated with the captive source in positive mode with following settings: mass range: 150–2200 *m*/*z*, 4 Hz, precursor acquisition control top20 (CID), capillary 1600 V, dry gas flow 3 l/min with 150°C, nanoBooster 0.2 bar.

LC–MS/MS data were analyzed with MaxQuant by searching the public Swissprot database with taxonomy *S. cerevisiae* and common contaminants. Carbamidomethylation on cysteine was entered as fixed modification, oxidation on methionine as variable modification. Detailed search criteria were used as follows: trypsin, max. missed cleavage sites: 2; search mode: MS/MS ion search with decoy database search included; precursor mass tolerance ±0.006 Da for Bruker data and ±4.5 ppm for Thermo data; product mass tolerance ±80 ppm for Bruker data and ±0.5 Da for Thermo data; acceptance parameters for identification: 1% PSM FDR; 1% protein FDR. In addition, a label free quantitation of each protein calculated from the areas under the curve of precursor ion intensity chromatograms was performed using MaxQuant (20) requiring a minimum of 2 ratio counts of quantified razor and unique peptides.

For a rough categorization, each detected protein was manually assigned to one of the following groups: 60S AFs, 90S AFs, 40S AFs, Npa1 complex members, H/ACA snoRNP components, C/D snoRNP components, 40S r-proteins, 60S r-proteins, others. For the comparison of Rbp95-TAP with Prp43-TAP the additional groups of G-patch proteins and mRNA splicing proteins were used. The determined label-free quantification (LFQ) intensity values were normalized so that the sum of intensities was 100% in each purification. All 0-values were replaced by 0.0001 to allow conversion into logarithmic values used for the graphical representation of the data. Then, the values of purifications to be compared were plotted against each other in Statgraphics 18 using logarithmic scaling.

The MS proteomics data have been deposited to the ProteomeXchange Consortium via the PRIDE (21) partner repository with the dataset identifier PXD030106. 

### Western blotting

Western blot analysis was performed using the following antibodies:

α-Rbp95 (1:5000) generated against full-length recombinant Rbp95 in rabbits (Eurogentec); α-CBP (1:5000; Merck-Millipore, cat.no. 07-482); α-HA (1:5000; Roche, cat. no. 12013819001); α-Dbp6 (1:10 000), α-Npa1 (1:5000), and α-Rsa3 (1:10 000) (10); α-Has1 (1:5000) and α-Nop7-Ytm1 (detecting both Nop7 and Ytm1; 1:5000), provided by Jesús de la Cruz; α-Arx1 (1:5000) and α-Nsa2 (1:5000), provided by Micheline Fromont-Racine; α-Nhp2 (1:5000) and α-Prp43 (1:4000), provided by Yves Henry; α-Nop1 (1:30 000) and α-Sof1 (1:300), provided by Ed Hurt; α-Enp1 (1:4000) and α-Rok1 (1:5000), provided by Katrin Karbstein; α-Rpl35 (1:35 000), provided by Matthias Seedorf; α-Noc1 (1:5000), α-Noc2 (1:5000) and α-Noc3 (1:5000), provided by Herbert Tschochner; α-Rpl3 (1:5000), provided by Jonathan Warner; α-Cic1/Nsa3 (1:5000), provided by Dieter Wolf; secondary α-rabbit horseradish peroxidase-conjugated antibody (1:15 000; Sigma, cat. no. A0545); secondary α-mouse horseradish peroxidase-conjugated antibody (1:10 000; Sigma, cat. no. NA931). Protein signals were visualized using the Clarity™ Western ECL Substrate Kit (Bio-Rad) and captured by ChemiDoc™ Touch Imaging System (Bio-Rad).

### LC–MS/MS for identification of proteins contained in gel bands

Protein-containing bands were excised from gels and digested with trypsin according to the method by Shevchenko *et al.* (22). Peptide extracts were dissolved in 0.1% formic acid and separated on a nano-HPLC system (Ultimate 3000™, LC Packings, Amsterdam, Netherlands). 70 μl samples were injected and concentrated on the loading column (LC Packings C18 Pep- Map™, 5 μm, 100 Å, 300 μm inner diameter × 1 mm) for 5 min using 0.1% formic acid as isocratic solvent at a flow rate of 20 μl/min. The column was then switched into the nanoflow circuit, and the sample was loaded on the nanocolumn (LC-Packings C18 PepMap™, 75 μm inner diameter × 150 mm) at a flow rate of 300 nl/min and separated using the following gradient: solvent A: water, 0.3% formic acid, solvent B: acetonitrile/water 80/20 (v/v), 0.3% formic acid; 0–5 min: 4% B, after 40 min 55% B, then for 5 min 90% B and 47 min re-equilibration at 4% B. The sample was ionized in a Finnigan nano-ESI source equipped with NanoSpray tips (PicoTip™ Emitter, New Objective, Woburn, MA, USA) and analyzed in a Thermo-Finnigan LTQ linear ion-trap mass-spectrometer (Thermo, San Jose, CA, USA). The MS/MS data were analyzed by searching the SwissProt public database with SpectrumMill Rev. 03.03.078 (Agilent, Darmstadt, GER) software.

### Fluorescence microscopy

Rbp95-GFP Nop58-mCherry yeast cells in logarithmic growth phase were imaged by fluorescence microscopy using a Leica DM6 B Microscope equipped with a ×100/1.4 Plan APO objective and narrow band GFP or RHOD ET filters. For imaging, the high-resolution DFC9000GT camera and the LASX premium software were used.

### Rapid depletion of Rbp95 via the auxin-inducible degron tag

Yeast cells expressing the plant F-box E3 ubiquitin ligase TIR1 and expressing Rbp95 with a C-terminal fused auxin-inducible degron (AID) tag from the genomic locus (23,24) were grown in 500 ml YPD medium at 30°C to an OD_600_ of 1 and then degradation of Rbp95-AID was induced by the addition of 500 μM of 3-indoleacetic acid (auxin; Sigma Aldrich; dissolved in 100% ethanol). Cells were harvested after 0, 15, 30, 60 and 120 min of auxin treatment. Additionally, 50 ml of the culture were incubated with an equal amount of ethanol only, serving as control, and cells were harvested after 120 min. All samples were further analyzed by western and northern blotting.

### Northern blotting

For total RNA isolation, cells were grown in 50 ml YPD medium at 30°C to an OD_600_ of ∼0.5 and harvested. Cells were resuspended in 200 μl lysis buffer containing 10 mM Tris–HCl (pH 7.5), 10 mM EDTA, and 0.5% SDS and mechanically lysed with 200 μl glass beads (0.5 mm diameter) for 3 min. After removal of glass beads, intact cells, and cell debris by centrifugation, supernatants were used for RNA isolation. For isolation of RNA from TAP purifications, 20 μl of the lysate as well as the total TAP eluate were adjusted to the same volume using TAP elution buffer. In both experiments (lysates or TAP-eluates), RNA was extracted by three phenol-chloroform-isoamyl alcohol (25:24:1) extractions and one chloroform-isoamyl alcohol (24:1) extraction. RNA was precipitated by addition of 1/10 volume of 3 M sodium acetate (pH 5.2), 2.5 volumes of 100% ethanol and 1 μl GlycoBlue™ coprecipitant (Invitrogen). After drying, RNA pellets were dissolved in nuclease-free water.

One to three μg of the RNA isolated from cell lysates or 50% of the sample from a TAP purification were separated on 1.6% MOPS-agarose gels containing 20 mM 3-(*N*-morpholino)-propanesulfonic acid (MOPS), 5 mM sodium acetate, 1 mM EDTA, 0.75% formaldehyde and ethidium bromide (pH 7.0), transferred overnight onto Hybond N^+^ nylon membranes (Amersham Biosciences) by capillary transfer and UV cross-linked to the membrane. Except for the probe E/C_2_ (27S A + B): 5′-GGC CAG CAA TTT CAA GTT A-3′, which was hybridized at 37°C, hybridization with the following 5′-^32^P-radiolabeled oligonucleotides was performed at 42°C overnight in buffer containing 0.5 M Na_2_HPO_4_ (pH 7.2), 7% SDS, and 1 mM EDTA: probe D/A_2_ (20S): 5′-GAC TCT CCA TCT CTT GTC TTC TTG-3′, probe A_2_A_3_ (35S, 32S/33S, 27SA_2_, 23S): 5′-TGT TAC CTC TGG GCC C-3′, probe 18S: 5′-GCA TGG CTT AAT CTT TGA GAC-3′, probe 25S: 5′-CTC CGC TTA TTG ATA TGC-3′. After four subsequent washing steps with buffer containing 40 mM Na_2_HPO_4_ (pH 7.2), 1% SDS, signals were detected by exposing X-ray films. Membranes were regenerated by washing in 1% SDS prior to hybridization.

### CRAC experiment

Yeast cells expressing Rbp95 fused at the C-terminus to the tripartite HTP tag (His6 tag-TEV protease cleavage site-Protein A tag) and the wild-type BY4742 strain (negative control) were grown at 30°C in 2.5 l of SDC-Trp medium to an OD_600_ of 0.6. Cells were irradiated with a megatron for 100 s with UV light at 254 nm and harvested. Cells were resuspended in TNM150 buffer (50 mM Tris–HCl (pH 7.8), 150 mM NaCl, 1.5 mM MgCl_2_, 0.1% NP-40, 5 mM β-mercaptoethanol) and lysed by mechanical disruption using zirconia beads. Cell lysates were mixed with 400 μl IgG Sepharose™ 6 Fast Flow slurry (GE Healthcare) pre-equilibrated with TNM150 buffer and incubated for two hours at 4°C on a stirring wheel. Beads were washed two times with TNM1000 buffer (50 mM Tris–HCl (pH 7.8), 1 M NaCl, 1.5 mM MgCl_2_, 0.1% NP-40, 5 mM β-mercaptoethanol) and two times with TNM150 buffer, then resuspended in 600 μl TNM150 buffer and transferred into Micro Bio-Spin 6 columns (Bio-Rad). Pre-ribosome elution from IgG Sepharose beads was achieved by incubation with 30 μl homemade GST-tagged TEV protease for two hours on a shaking table at 16°C. TEV eluates (about 650–700 μl) were partially digested for 5 min at 37°C with 1.4 μl of RNace-IT (Agilent) diluted to 1:50 in TNM150 buffer and the reactions were stopped using 0.4 g guanidine hydrochloride. The resulting samples were supplemented with 300 mM NaCl and 10 mM imidazole and incubated overnight at 4°C on a stirring wheel with 50 μl Ni-NTA agarose resin slurry (QIAGEN) pre-equilibrated with wash buffer I (50 mM Tris–HCl (pH 7.8), 300 mM NaCl, 10 mM imidazole, 6 M guanidine hydrochloride, 0.1% NP-40, 5 mM β-mercaptoethanol). Ni-NTA beads were then washed two times with wash buffer I, three times with 1× PNK buffer (50 mM Tris–HCl (pH 7.8), 10 mM MgCl_2_, 0.5% NP-40, 5 mM β-mercaptoethanol) and transferred into Pierce Spin Columns (Thermo Scientific). RNAs retained on the Ni-NTA beads were dephosphorylated for 30 min at 37°C using TSAP (Promega) in 1× PNK buffer in a total volume of 80 μl in the presence of 80 units of RNasin ribonuclease inhibitor (Promega). Beads were washed once with wash buffer I to inactivate TSAP and three times with 1× PNK buffer. The miRCat-33 linker (5′-AppTGG AAT TCT CGG GTG CCA AG/ddC/-3′) was ligated to the 3′ end of the RNAs on the Ni-NTA beads with 800 units of T4 RNA ligase 2 truncated K227Q (New England Biolabs) in 1 x PNK buffer / 16.67% PEG 8000 in the presence of 80 units RNasin in a total volume of 80 μl. The ligation reaction was incubated for five hours at 25°C. Beads were washed once with wash buffer I to inactivate the RNA ligase and 3 times with 1× PNK buffer. The 5′ ends of the RNAs were then radiolabeled by phosphorylation in reactions containing 1× PNK buffer, 40 μCi of ^32^P-ɣ ATP and 20 units of T4 PNK (Sigma) in a total volume of 80 μl. The reactions were incubated at 37°C for 40 min. To ensure all RNAs get phosphorylated at the 5′ end for downstream ligation of the 5′ linker, 1 μl of 100 mM ATP was added to the reaction mix, which was incubated for another 20 min at 37°C. Beads were washed once with wash buffer I to inactivate the kinase and four times with 1 x PNK buffer. Solexa linkers L5Aa (5′-invddT-ACA CrGrAr CrGrCr UrCrUr UrCrCr GrArUr CrUrNr NrNrUr ArArG rC-OH-3′) and L5Ab (5′-invddT-ACA CrGrAr CrGrCr UrCrUr UrCrCr GrArUr CrUrNr NrNrAr UrUrAr GrC-OH-3′) were ligated to the 5′ end of the RNAs retained on the Ni-NTA beads for the BY4742 and Rbp95-HTP samples, respectively. Ligation reactions contained 1× PNK buffer, 1.25 μM of the relevant Solexa linker, 1 mM ATP, 40 units of T4 RNA Ligase 1 (New England Biolabs) in a total volume of 80 μl. The reactions were incubated overnight at 16°C. Beads were washed three times with wash buffer II (50 mM Tris–HCl (pH 7.8), 50 mM NaCl, 10 mM imidazole, 0.1% NP-40, 5 mM β-mercaptoethanol). The material retained on the beads was then eluted using two times 200 μl elution buffer (50 mM Tris-HCl (pH 7.8), 50 mM NaCl, 150 mM imidazole, 0.1% NP-40, 5 mM β-mercaptoethanol). Eluates were precipitated with TCA (20% final concentration) in the presence of 30 μg of glycogen (Roche) to favor precipitation. The precipitated material was resuspended in NuPAGE™ LDS sample buffer (Invitrogen) with reducing agent (Invitrogen), heated 10 min at 65°C, loaded on NuPAGE™ 4–12% Bis–Tris gels (Invitrogen) and run in 1× MOPS SDS running buffer (Invitrogen). The material was then transferred onto Amersham Protran Nitrocellulose Blotting Membrane (GE Healthcare), using a transfer buffer containing 1× NuPAGE (Invitrogen) and 20% MeOH, for two hours at 25 V and 4°C. For the Rbp95-HTP sample, the area of the membrane containing a radioactive signal at the expected size of Rbp95 protein was excised. A membrane area at the same size was excised in the BY4742 sample lane. Membranes were soaked in 400 μl wash buffer II supplemented with 1% SDS, 5 mM EDTA and proteins were degraded using 100 μg proteinase K (Sigma) and incubation for two hours at 55°C. RNA was extracted with phenol:chloroform:isoamyl alcohol (25:24:1), and then precipitated by addition of 1:10 volume of 3 M sodium acetate (pH 5.2), 2.5 volumes of 100% ethanol and 20 μg of glycogen. Dried RNA pellets were dissolved in ultrapure MilliQ H_2_O. Synthesis of cDNAs was performed using SuperScript III reverse transcriptase (Thermo Fischer Scientific) and oligonucleotide miRcatRT (5′-CCT TGG CAC CCG AGA ATT-3′). The resulting cDNAs were PCR-amplified using LA Taq DNA polymerase (TaKaRa) and primers P5F (5′-AAT GAT ACG GCG ACC ACC GAG ATC TAC ACT CTT TCC CTA CAC GAC GCT CTT CCG ATC T-3′) and P3R (5′-CAA GCA GAA GAC GGC ATA CGA GAT CCT TGG CAC CCG AGA ATT CC-3′). The resulting PCR products were purified by phenol:chloroform:isoamyl alcohol extraction and ethanol precipitation. After agarose gel electrophoresis (agarose ‘small fragments’, Eurogentec) run in 1× TBE buffer and stained with SYBR Safe DNA gel stain (Invitrogen), DNA fragments ranging in size between 150 and 250 base pairs were gel purified using MinElute PCR Purification Kit (QIAGEN). Concentration of the final DNA samples was measured using Qubit™ dsDNA HS Assay Kit (Invitrogen) and a Qubit™ fluorometer (Thermo Fischer Scientific) and the samples were sent to the GeT-PlaGe Genotoul facility for Illumina sequencing.

### Deep-sequencing and computational analyses

Sequencing was performed using an Illumina HiSeq system. Barcodes, adapters and low-quality reads were eliminated using Flexbar (http://sourceforge.net/projects/flexbar/). Remaining reads were aligned to the yeast genome using Novoalign (http://www.novocraft.com). Downstream analyses including the pileups were performed using the pyCRAC tool suite (http://sandergranneman.bio.ed.ac.uk/Granneman_Lab/pyCRAC_software.html). Hits repartition per million of sequences were produced using pyReadCounter, py—m 1 000 000 option. Different pileups of hits for each gene were obtained using pyPileup.py—L 50—limit = 100 000 options. NGS analysis files of raw and processed data were deposited in the Gene Expression Omnibus database under the accession number GSE189589.

### Purification of recombinant Rbp95

*RBP95* or sub-fragments thereof were cloned into a pETDuet-1 vector (Novagen), enabling the expression of N-terminally His6-tagged Rbp95, and *Escherichia coli* Rosetta (DE3) (Novagen) was transformed with the plasmids. Cells were cultured in 1 l LB medium containing 50 μg/ml ampicillin at 37°C to an OD_600_ of 0.3–0.4. Protein expression was induced with 0.3  mM isopropyl β-d-thiogalactoside (IPTG), and cultures were shifted to 16°C for 20  h. Cell pellets were resuspended in a 1.5-fold volume of lysis buffer containing 50 mM Tris–HCl (pH 7.5), 500 mM NaCl, 5 mM MgCl_2_, 7% glycerol, 0.05% Tween 20, 1 mM dithiothreitol (DTT), 1× HP protease inhibitor mix (Serva), 1 mM phenylmethylsulphonyl fluoride (PMSF), and 1 mg/ml lysozyme, and lysed by sonication. Cell lysates were centrifuged at 44 000 g at 4°C for 45 min to remove insoluble material. Then, imidazole was added to a final concentration of 15 mM to the supernatants, which were incubated for 2 h under rotation at 4°C with Ni-NTA agarose beads (Qiagen). Beads were then washed five times with ∼1 ml washing buffer (50 mM Tris–HCl (pH 7.5), 500 mM NaCl, 5 mM MgCl_2_, 7% glycerol, 0.05% Tween 20, 1 mM DTT), additionally containing 15 mM imidazole, and then two times with washing buffer containing 50 mM imidazole.

His6-Rbp95 protein and truncations thereof were then eluted at 4°C under rotation in washing buffer containing 500 mM imidazole and eluates were subjected to a buffer exchange into a buffer containing 75 mM NaCl, 50 mM Tris–HCl (pH 7.5), 5 mM MgCl_2_, 3.5% glycerol, 0.05% Tween 20, and 1 mM DTT using Zeba Spin Desalting Columns (Thermo Fisher Scientific). To remove non-specifically co-purified RNA from the purifications, eluates were then loaded onto a 1 ml HiTrapHeparinHP column (GE Healthcare). The column was run on an ÄKTA FPLC system at 0.5 ml/min flow rate in buffer containing 50 mM Tris–HCl (pH 7.5), 5 mM MgCl_2_, 3.5% glycerol, 0.05% Tween 20 and 1 mM DTT, employing a 20 ml salt gradient from 75 to 1,500 mM NaCl. 1 ml fractions were collected. Fractions containing the respective eluted proteins were then concentrated using Amicon Ultra4 Centrifugal Filters10k (Millipore) to a volume of 300 μl and then subjected to a buffer exchange to a buffer containing 50 mM Tris–HCl (pH 7.5), 150 mM NaCl, 5 mM MgCl_2_, 7% glycerol, 0.05% Tween 20 and 1 mM DTT using Zeba Spin Desalting Columns.

### Electrophoretic mobility shift assays (EMSAs)

5′Cy5-labeled RNA-oligonucleotides helix H95 (5′-AAA GGG AAU UGA ACU UAG UAC GAG AGG AAC AGU UCA UUU CCC-3′), helix H45 (5′-AAA GGG CGU CAG GGU UGA UAU GAU GCC CUG ACG CCC-3′), and poly(A)_42_ were ordered from Eurofins. H95 and H45 oligonucleotides were annealed using 3 min denaturation at 95°C and slow cooling (∼20 min) to 4°C prior to using them in the assays. For the EMSAs with full-length Rbp95 in Figure 6A, Cy5-labeled RNA-oligonucleotides (5 nM concentration) were incubated for 30 min at RT with an increasing molar excess (0-, 1.5-, 3.75-, 7.5- and 15-fold) of purified Rbp95 in a total volume of 15 μl buffer containing 25 mM Tris–HCl (pH 7.5), 75 mM NaCl, 2.5 mM MgCl_2_, 3.5% glycerol, 0.025% Tween 20 and 0.5 mM DTT. Subsequently, 3.75 μl loading buffer (containing 2.5× Novex™ TBE running buffer (Thermo Fisher Scientific) and 15% Ficoll type 400 (Serva)) was added and samples were loaded onto Novex™ 4–20% TBE gels (Thermo Fisher Scientific) and run in precooled 0.5 x TBE running buffer at 200 V, 50 min at RT. Cy5-signals were subsequently detected on a Bio-Rad ChemidocMP imaging system. For the competition experiments, 5 nM Cy5-labeled H95-oligo was incubated with a 15-fold excess of full-length Rbp95 and, in addition, 0-, 20-, 50- or 100-fold excess of unlabeled oligo H95 or poly(A) (relative to labeled oligo) was used. For the EMSAs with truncated Rbp95 fragments in Figures 6D and 5 nM Cy5-labeled RNA-oligonucleotides were incubated with a 100- or 500-fold molar excess of Rbp95. For the three fragments for which a shift was already observed at 100-fold molar excess (1–85, 84-end, full length), additionally also lower concentrations were tested (2.5-, 10-, 100- or 500-fold; Supplementary Figure S7).

### Identification of protein neighborhoods by TurboID-based proximity labeling

Plasmids expressing C-terminally TurboID-tagged bait proteins under the control of the copper-inducible *CUP1* promoter were transformed into the wild-type strain YDK11-5A. Transformed cells were grown at 30°C in 100 ml SDC-Leu medium, prepared with copper-free yeast nitrogen base (FORMEDIUM), to an OD_600_ between 0.4 and 0.5. Then, copper sulfate, to induce expression from the *CUP1* promoter, and freshly prepared biotin (Sigma-Aldrich) were added to a final concentration of 500 μM, and cells were grown for an additional hour, typically reaching a final OD_600_ between 0.6 and 0.8, and harvested by centrifugation at 4000 rpm for 5 min at 4°C. Then, cells were resuspended in 1 ml ice-cold lysis buffer (LB: 50 mM Tris–HCl (pH 7.5), 150 mM NaCl, 1.5 mM MgCl_2_, 0.1% SDS and 1% Triton X-100) containing 1 mM PMSF, transferred to 2 ml safe-lock tubes, pelleted by centrifugation, frozen in liquid nitrogen, and stored at -80°C. Extracts were prepared, upon resuspension of cells in 400 μl lysis buffer containing 0.5% sodium deoxycholate and 1 mM PMSF (LB-P/D), by glass bead lysis with a Precellys 24 homogenizer (Bertin Technologies) set at 5000 rpm using a 3 × 30 s lysis cycle with 30 s breaks in between at 4°C. Lysates were transferred to 1.5 ml tubes. For complete extract recovery, 200 μl LB-P/D were added to the glass beads and, after brief vortexing, combined with the already transferred lysate. Cell lysates were clarified by centrifugation for 10 min at 13 500 rpm at 4°C, transferred to a new 1.5 ml tube, and the volume was completed to around 800 μl by the addition of around 200 μl LB-P/D. Total protein concentration in the clarified cell extracts was determined with the Pierce™ BCA Protein Assay Kit (Thermo Scientific) using a microplate reader (BioTek 800 TS). To reduce non-specific binding, 100 μl of Pierce High Capacity Streptavidin Agarose Resin (Thermo Scientific) slurry, corresponding to 50 μl of settled beads, were transferred to a 1.5 ml safe-lock tube, blocked by incubation with 1 ml LB containing 3% BSA for 1 h at RT, and then washed four times with 1 ml LB. For affinity purification of biotinylated proteins, 2 mg of total protein in an adjusted volume of 800 μl LB-P/D was added to the blocked and washed streptavidin beads, and binding was carried out for 1 h at RT on a rotating wheel. Beads were then washed once for 5 min with 1 ml of wash buffer (50 mM Tris–HCl (pH 7.5), 2% SDS), five times with 1 ml LB, and finally five times with 1 ml ABC buffer (100 mM ammonium bicarbonate (pH 8.2)). Bound proteins were eluted by two consecutive incubations with 30 μl 3× SDS sample buffer, containing 10 mM biotin and 20 mM DTT, for 10 min at 75°C. The eluates were combined in one 1.5 ml safe-lock tube and stored at –20°C. Upon reduction with DTT and alkylation with iodoacetamide, samples were separated on NuPAGE 4–12% Bis–Tris gels (Invitrogen), run in NuPAGE 1× MES SDS running buffer (Novex) at 200 V for a total of 12 min. The gels were incubated with Brilliant Blue G Colloidal Coomassie (Sigma-Aldrich) until staining of proteins was visible. Each lane was cut, from slot to the migration front, into three gel pieces of equal lengths that were, upon their fragmentation into smaller pieces, transferred into separate 1.5 ml low binding tubes.

Gel pieces were covered with 100–150 μl of ABC buffer, prepared in HPLC grade H_2_O, and incubated for 10 min at RT in a thermoshaker set to 1000 rpm. Then, gel pieces were covered with 100–150 μl of HPLC grade absolute EtOH and incubated for 10 min at RT in a thermoshaker set to 1000 rpm. These two wash steps were repeated two more times. For in-gel digestion of proteins, gel pieces were covered with 120 μl of ABC buffer containing 1 μg Sequencing Grade Modified Trypsin (Promega) and incubated overnight at 37°C with shaking at 1000 rpm. To stop the digestion and recover the peptides, 50 μl of a 2% trifluoroacetic acid (TFA) solution was added, and, after a 10 min incubation at RT with shaking at 1000 rpm, the supernatant was transferred to a new 1.5 ml low binding tube. The gel pieces were then incubated, again for 10 min at RT with shaking at 1000 rpm, another two times with 100–150 μl EtOH, and these two supernatants were combined with the first supernatant. Finally, using a SpeedVac, the organic solvents were evaporated and the volume was reduced to around 50 μl. Then, 200 μl of buffer A (0.5% acetic acid) were added, and the samples were applied to C18 StageTips (25), equilibrated with 50 μl of buffer B (80% acetonitrile, 0.3% TFA) and washed twice with 50 μl of buffer A, for desalting and peptide purification. StageTips were washed once with 100 μl of buffer A, and the peptides were eluted with 50 μl of buffer B. The solvents were completely evaporated using a SpeedVac, and the peptides were resuspended in 20 μl of buffer A*/A (30% buffer A* (3% acetonitrile, 0.3% TFA) / 70% buffer A) and stored at –80°C.

LC–MS/MS measurements were performed on a Q Exactive Plus (Thermo Scientific) coupled to an EASY-nLC 1200 nanoflow-HPLC (Thermo Scientific). HPLC-column tips (fused silica) with 75 μm inner diameter were self-packed with ReproSil-Pur 120 C18-AQ, 1.9 μm particle size (Dr. Maisch GmbH) to a length of 20 cm. Samples were directly applied onto the column without a pre-column. A gradient of A (0.1% formic acid in H_2_O) and B (0.1% formic acid in 80% acetonitrile in H_2_O) with increasing organic proportion was used for peptide separation (loading of sample with 0% B; separation ramp: from 5–30% B within 85 min). The flow rate was 250 nl/min and for sample application 600 nl/min. The mass spectrometer was operated in the data-dependent mode and switched automatically between MS (max. of 1 × 10^6^ ions) and MS/MS. Each MS scan was followed by a maximum of ten MS/MS scans using normalized collision energy of 25% and a target value of 1000. Parent ions with a charge state form *z* = 1 and unassigned charge states were excluded for fragmentation. The mass range for MS was *m*/*z* = 370–1750. The resolution for MS was set to 70 000 and for MS/MS to 17 500. MS parameters were as follows: spray voltage 2.3 kV, no sheath and auxiliary gas flow, ion-transfer tube temperature 250°C.

The MS raw data files were analyzed with the MaxQuant software package version 1.6.2.10 (26) for peak detection, generation of peak lists of mass-error-corrected peptides, and database searches. The UniProt *Saccharomyces cerevisiae* database (version March 2016), additionally including common contaminants, trypsin, TurboID, and GFP, was used as reference. Carbamidomethylcysteine was set as fixed modification and protein amino-terminal acetylation, oxidation of methionine, and biotin were set as variable modifications. Four missed cleavages were allowed, enzyme specificity was Trypsin/P, and the MS/MS tolerance was set to 20 ppm. Peptide lists were further used by MaxQuant to identify and relatively quantify proteins using the following parameters: peptide and protein false discovery rates, based on a forward-reverse database, were set to 0.01, minimum peptide length was set to seven, and minimum number of unique peptides for identification and quantification of proteins was set to one. The ‘match-between-run’ option (0.7 min) was used.

For quantification, missing iBAQ (intensity-based absolute quantification) values in the control purification from cells expressing the NLS-GFP-TurboID bait were imputed in Perseus (27). For normalization of intensities in each independent purification, iBAQ values were divided by the median iBAQ value, derived from all nonzero values, of the respective purification. To calculate the enrichment of a given protein compared to its abundance in the control purification, the normalized iBAQ values were log_2_ transformed and those of the control purification were subtracted from the ones of each respective bait purification. The normalized iBAQ value (log_10_ scale) of each protein detected in a given bait purification was plotted against its relative abundance (log2-transformed enrichment) compared to the control purification.

### Software for heatmap generation, sequence alignment, visualization of structural data

The heatmap was generated in Genesis (28). Sequence alignments were performed with Clustal Omega (www.ebi.ac.uk) (29) and visualized using Jalview (www.jalview.org) (30). (Pre-)ribosome structures were visualized in PyMOL 2.2.3 (Schrödinger).

## RESULTS

### Ycr016w/Rbp95 participates in the early, nucleolar stages of ribosome assembly

**Figure 1.**
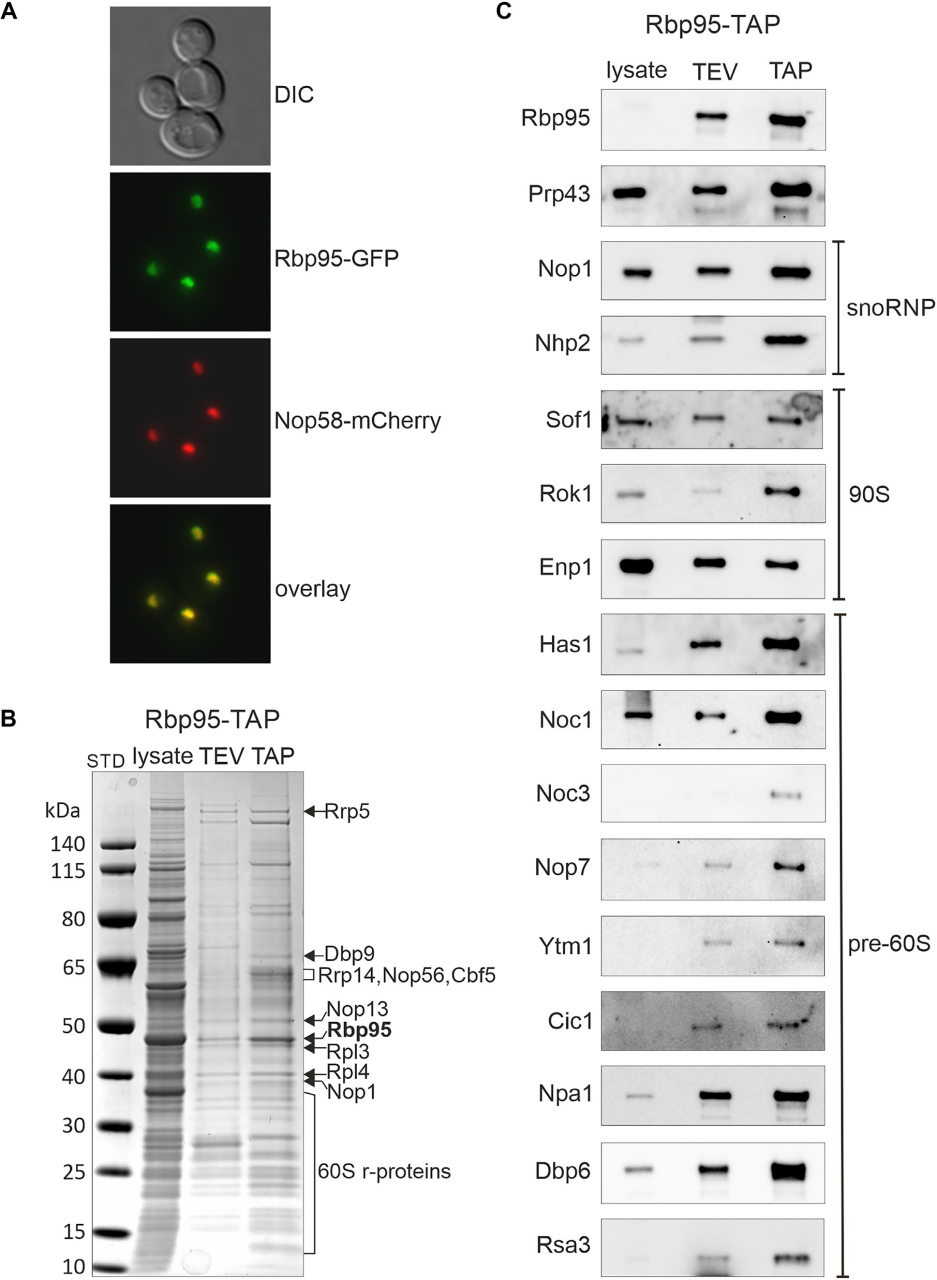
Ycr016w/Rbp95 is a novel ribosome AF. (**A**) Rbp95-GFP is localized in the nucleolus. DIC: differential interference contrast image. Nop58-mCherry was used as nucleolar marker that colocalizes with Rbp95-GFP. (**B**) Ribosome AFs co-purify with Rbp95-TAP. Lysate, TEV-eluate, and final TAP eluate of a Rbp95-TAP purification were separated on a 4–12% NuPAGE gradient gel, which was stained with Coomassie blue. Proteins identified by mass spectrometry are indicated. STD, molecular weight standard. (**C**) Samples prepared as in (B) were subjected to SDS-PAGE and western blotting using antibodies recognizing the indicated AFs, which are characteristic components of snoRNPs or the indicated pre-ribosomal particles.

**Figure 2.**
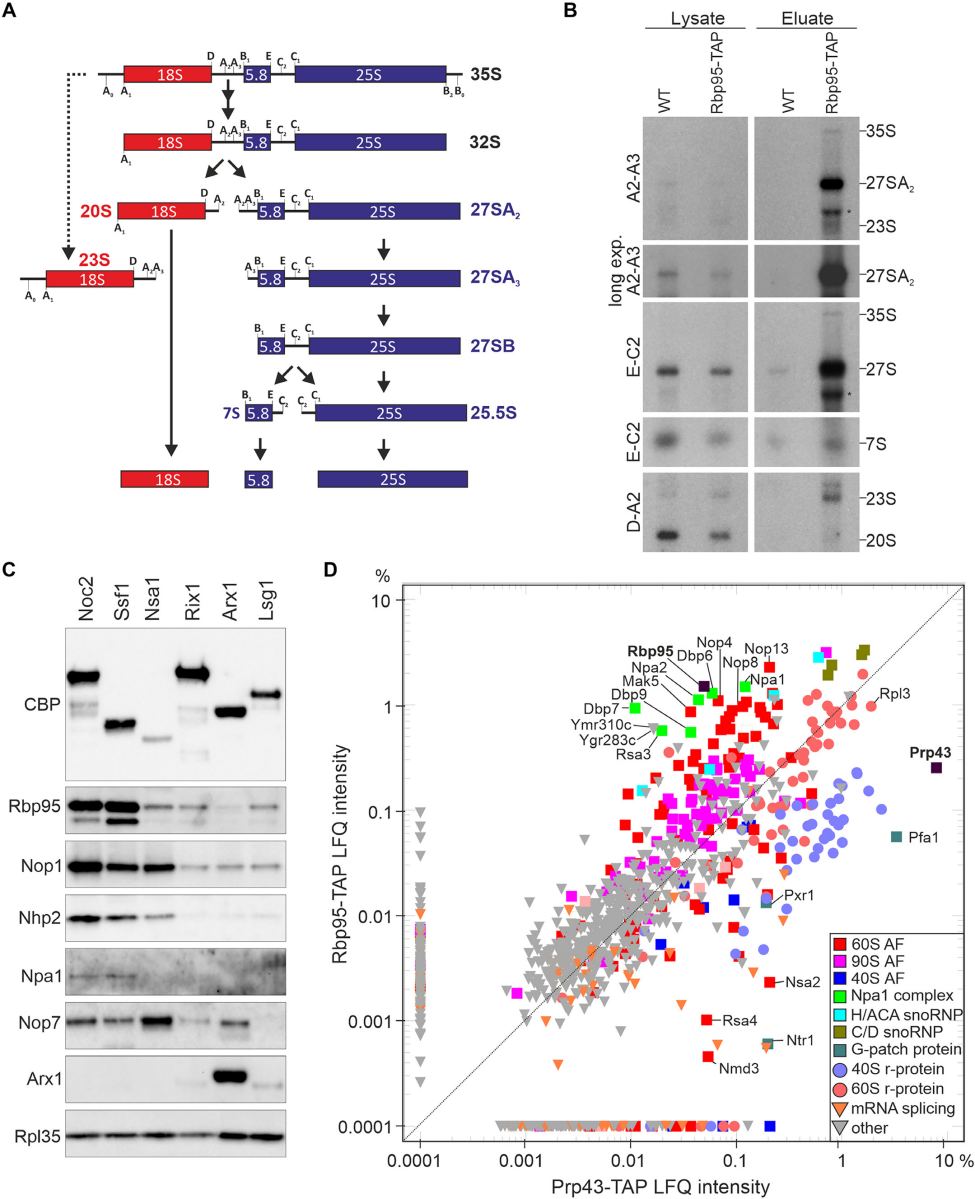
Rbp95 is associated with 90S particles and early pre-60S particles containing the Npa1-complex. (**A**) Simplified overview of the yeast pre-rRNA processing pathway showing the main intermediates as well as the 23S pre-rRNA, which accumulates upon inhibited or delayed processing at the early cleavage sites A_0_, A_1_ and A_2_. The main pre-rRNA cleavage sites are indicated. (**B**) Detection of pre-rRNA species by northern blotting analysis in the lysate or TAP eluate of strains expressing untagged (WT) or TAP-tagged Rbp95 (Rbp95-TAP). The detected pre-rRNA species are indicated on the right and the used probes on the left. The names of the probes indicate between which processing sites, shown in (A), they hybridize to the pre-rRNA. The bands labeled by an asterisk detected below the 27S pre-rRNA with the A2–A3 and the E-C2 probe likely originate from non-specific hybridization with contaminating mature 25S rRNA. (**C**) Eluates obtained by TAP purification of different intermediates of the pre-60S maturation pathway, arranged from early (left) to late (right) pre-60S particles, were analyzed by western blotting using antibodies recognizing the indicated snoRNP components and AFs as well as the large subunit r-protein Rpl35. CBP, calmodulin binding protein (moiety of the TAP-tag). (**D**) Label-free semi-quantitative mass spectrometry and comparison of LFQ (label-free quantification) intensities between Rbp95-TAP and Prp43-TAP eluates (in percent relative to the total intensity of proteins detected in the respective purification). Proteins particularly enriched in one of the purifications, as well as Rpl3, are labeled. Bait proteins are indicated with a black square and labeled in bold letters.

In the course of our work on Prp43, an RNA helicase with a dual function in pre-mRNA splicing and ribosome biogenesis, we noticed that the so far uncharacterized protein Ycr016w, subsequently termed Rbp95 (for rRNA-binding protein helix H95; see below), was co-enriched in affinity purifications of both Pxr1/Gno1 and Pfa1, two specific activators of Prp43 during distinct ribosome biogenesis steps (31,32) (Supplementary Figure S1). Moreover, Rbp95 was previously reported to also co-purify with Prp43 (33), a finding that could be confirmed by our experiments (Figure 2D and data not shown). As however neither yeast two-hybrid (Y2H) assays nor *in vitro* binding assays with recombinant proteins provided evidence for a physical interaction of Rbp95 with Prp43 or G-patch proteins (data not shown), we speculate that Rbp95 is not directly interacting with these proteins but is instead present in the same pre-ribosomal particles as Prp43, Pxr1 and Pfa1. Moreover, we observed that Rbp95-GFP colocalized with the nucleolar marker Nop58-mCherry and was hence exclusively localized in the nucleolus (Figure 1A), confirming previous large-scale data (34). Additionally, *RBP95* was reported to be co-regulated with ribosome biogenesis genes (35). Hence, both previous data and our findings suggest that Rbp95 functions in nucleolar stages of ribosome biogenesis.

To better define the stage of ribosome biogenesis in which Rbp95 functions, we performed Rbp95-TAP purifications, analyzed the eluates by Coomassie blue staining, excised a few selected prominent bands, and identified the contained proteins by mass spectrometry (Figure 1B). The results revealed that multiple r-proteins of the large subunit were co-purified. In addition, the C/D box snoRNP proteins Nop1 and Nop58, the H/ACA box snoRNP protein Cbf5, and the early pre-60S factors Rrp5, Dbp9, Rrp14 and Nop13 were identified. We also analyzed the Rbp95-TAP purified particles by western blotting. These analyses revealed that not only Prp43 and snoRNP proteins, but also 90S and pre-60S AFs were present in the Rbp95-TAP eluate (Figure 1C). Based on the nucleolar localization of Rbp95 and its association with pre-ribosomal particles, we conclude that Rbp95 is a novel ribosome AF participating in the early, nucleolar stages of ribosome assembly.

### Rbp95 is bound to early, Npa1 complex-containing pre-60S particles

To pinpoint the pre-ribosomal particles containing Rbp95 more precisely, we extracted RNAs co-purified with Rbp95-TAP and performed northern blotting to detect intermediates of the pre-rRNA processing pathway (Figure 2A). Several pre-rRNAs were co-purified with Rbp95, including small amounts of the 35S pre-rRNA, which is present in 90S pre-ribosomal particles (Figure 2B). This further confirms that Rbp95 binds already at the stage of 90S particles, in line with the detection of 90S factors in the Rbp95-TAP purification (Figure 1B, C). Moreover, also the 23S rRNA was co-purified to some extent with Rbp95-TAP (Figure 2B); however, the nature and function of the 23S rRNA is not fully understood so far. It was previously interpreted to be an aberrant, dead-end intermediate (36,37), but is considered in alternative models as a functional processing intermediate that is formed in the post-transcriptional rRNA processing pathway, in which synthesis of 35S pre-rRNA is completed before pre-rRNA processing starts (1). The pre-rRNA species that was however most enriched in the Rbp95-TAP purification was the 27SA_2_ pre-rRNA, a characteristic component of early pre-60S particles. Additionally, a strong signal was also obtained with a probe detecting not only 27SA_2_ pre-rRNA, but also the later 27SA_3_ and 27SB pre-rRNAs. As this ‘total-27S’ signal was clearly stronger than the 27SA_2_ signal, when compared to the 35S pre-rRNA detected by both probes, 27S species later than 27SA_2_ have to be present in the purification. Quantification of the signals indicated that ∼3.8% of Rbp95-associated particles contained the 35S pre-rRNA, ∼77% the 27SA_2_ pre-rRNA, and ∼19.2% later 27S pre-rRNA species. This suggests that Rbp95 is mainly bound to early pre-60S particles and is released soon after conversion of the 27SA_2_ pre-rRNA to later forms.

To further define the stages of pre-60S maturation in which Rbp95 participates, we affinity purified a series of different pre-60S particles, ranging from early nucleolar to late cytoplasmic particles, and detected Rbp95 by western blotting (Figure 2C). Rbp95 co-purified most strongly with Noc2-TAP and Ssf1-TAP. Noc2 and its partner Noc1, join at the stage of 90S particles and are present in early pre-60S particles. While Noc1 is released at the stage of early pre-60S particles, Noc2 then forms a complex with a new partner Noc3 and dissociates after formation of 27SB pre-rRNAs (1,38). Ssf1 binds later than Noc2 and is released at a very early pre-60S stage, likely around the time when Noc1 is replaced by Noc3 (39). In contrast, only minor amounts of Rbp95 were co-purified with Nsa1-TAP (note that similar amounts of pre-60S particles were loaded, as indicated by the similar levels of the early-binding 60S r-protein Rpl35 (40)) or later pre-60S particle baits (Rix1-TAP, Arx1-TAP or Lsg1-TAP) (Figure 2C). Although Nsa1 is predominantly localized in the nucleolus like Ssf1, slightly later pre-60S particles are co-purified with Nsa1 compared to Ssf1 (39). Interestingly, the association pattern of Rbp95 was very similar to the one of the early pre-60S factor Npa1, which was only detected in Noc2- and Ssf1-TAP purifications, but not in the Nsa1-TAP purification. In contrast, the H/ACA snoRNP protein Nhp2 and the C/D box snoRNP protein Nop1 remained bound to pre-60S particles a bit longer and were also detected in the Nsa1-TAP purification (although Nhp2 was reduced).

To characterize the protein interactome of Rbp95 in a more quantitative way, we compared the proteins co-purified with Rbp95-TAP and Prp43-TAP by label-free semi-quantitative mass spectrometry (Figure 2D, Supplementary Table S4). Interestingly, although a similar set of proteins was co-purified with both baits, their quantities strongly varied. Prp43 is known to act not only in 60S subunit biogenesis, but also in 40S subunit biogenesis and pre-mRNA splicing (41). Therefore, it is not surprising that 40S r-proteins and pre-40S AFs, as well as splicing factors, were much more represented in the Prp43-TAP purification compared to the Rbp95-TAP purification. In contrast, pre-60S AFs were in relation more strongly co-enriched with Rbp95-TAP than with Prp43-TAP. The same was true for C/D- and even more so for H/ACA-snoRNP components, which were more represented in the Rbp95-TAP compared to the Prp43-TAP purification. The proteins that were however most strongly enriched in Rbp95-TAP were Npa1, Npa2, Dbp6, Dbp7, Dbp9, Rsa3 and Nop8, all belonging to a sub-complex of early pre-60S particles termed the Npa1 complex (10,42–44). A few other proteins involved in early pre-60S maturation (Mak5, Nop4, Nop13) and two mostly uncharacterized proteins (Ygr283c and Ymr310c), which were recently renamed Upa1 and Upa2, and reported to be components of early pre-60S particles (45), were enriched as well. Together, our results indicate that Rbp95 is bound to 90S and early pre-60S particles, similar to Npa1 complex members and the early pre-60S factors Mak5, Nop4 and Nop13.

### A synthetic lethal screen reveals a strong functional connection between Rbp95 and members of the Npa1 complex network

To obtain better insight into the functional role of Rbp95, whose deletion does not result in any apparent growth defect at any temperature tested (Supplementary Figure S2), we performed a synthetic lethal (SL) screen with a Δ*rbp95* mutant (see Materials and Methods section for experimental details). In total, 12 independent mutants were isolated that exhibited either an SL phenotype or a synthetic growth defect (also referred to as synthetic enhancement (SE) phenotype) in combination with the deletion of *RBP95*.

**Figure 3.**
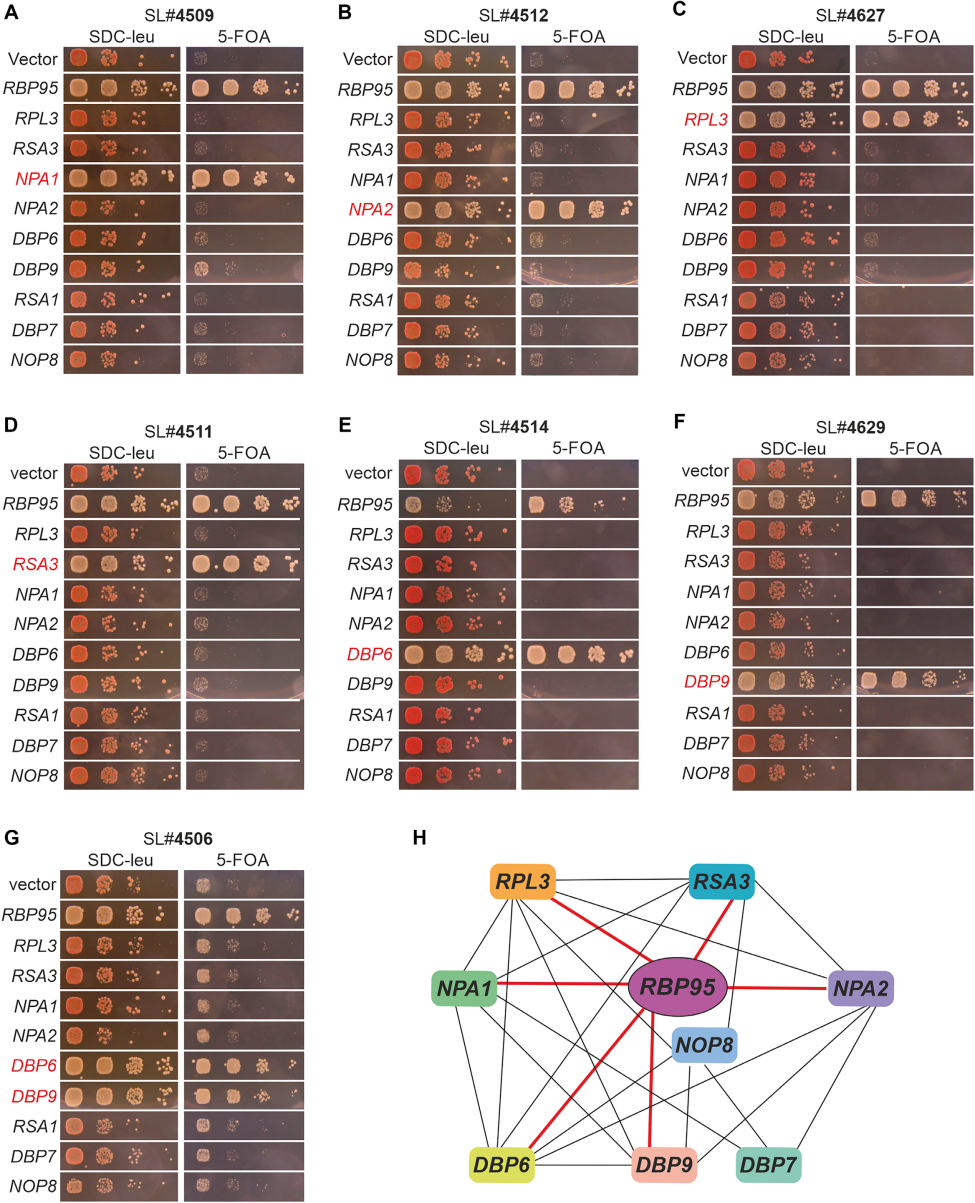
Rbp95 is functionally connected to Rpl3 and Npa1 network members. (**A–G**) Mutants isolated in the SL screen were transformed with vector, *RBP95*-containing plasmid, or plasmids harboring the indicated genes (*RPL3* and other genes from the *NPA1* genetic network). While transformation with empty vector resulted in no complementation, as illustrated by the red colonies on SDC-Leu plates and the inviability or severe growth defect on 5-FOA-containing plates, transformation with the *RBP95-*containing plasmid led to white (or red/white sectoring) colonies and restoration of growth on 5-FOA-containing plates. Genes other than *RBP95* complementing the SL or SE phenotype are highlighted in red. (**H**) Genetic network around Npa1 complex members. Genetic interactions revealed in this study (thick red lines) and previously described ones (thin black lines) are schematically summarized.

After transformation of one of these mutants (#4509) with a yeast genomic library, we found that its SE phenotype could be complemented by *NPA1* (Figure 3A). PCR-amplification and sequencing of the genomic locus of this mutant confirmed that *NPA1* indeed contained a frame-shift mutation, resulting in a G1729 > R and a I1730 > N exchange followed by a pre-mature stop codon and, thus, leading to a truncated protein lacking the 34 C-terminal residues. Next, we asked whether the other mutants isolated in the screen also carried mutations in *NPA1*. To address this, we transformed the *NPA1*-containing plasmid that had complemented mutant #4509 into the remaining mutants. None of them was complemented by *NPA1*, suggesting that their SL phenotypes are caused by mutations in other genes.

*NPA1* is part of a genetic interaction network including *NPA2*, *DBP6*, *RSA3*, *NOP8*, *DBP7*, *DBP9, RSA1* and *RPL3* (16,42,46–49). Therefore, we speculated that one or several of these genes might be mutated in our remaining SL strains. To address this, we transformed all of our SL mutants with centromeric plasmids carrying either *NPA1*, *NPA2*, *DBP6*, *RSA3*, *NOP8*, *DBP7*, *DBP9*, *RSA1* or *RPL3*. Indeed, one of the mutants (#4512) was complemented by *NPA2* (Figure 3B), two mutants (#4510 and #4627) by *RPL3* (Figure 3C and data not shown), two mutants (#4511 and #5009) by *RSA3* (Figure 3D and data not shown), one mutant (#4514) by *DBP6* (Figure 3E), and two mutants (#4629 and #5010) by *DBP9* (Figure 3F and data not shown). In all these cases, sequencing of the genomic locus confirmed that the complementing genes were mutated in the respective SL mutants (Supplementary Table S3). Moreover, mutant #4506 was complemented by both *DBP6* and *DBP9* (Figure 3G). Previous studies indicated that *DBP9* can act as a dosage suppressor of some *dbp6* mutants (48). Indeed, sequencing of both genes in this mutant revealed that only *DBP6* was mutated, while the *DBP9* wild-type sequence was present, suggesting that, similar to the previous observation, increased dosage of *DBP9* suppressed this *dbp6* allele (Supplementary Table S3). Last but not least, two mutants isolated in the screen were not complemented by any of the tested genes and remain unidentified. In summary, our screen revealed genetic interactions of *RBP95* with *NPA1*, *NPA2*, *DBP6*, *DBP9*, *RSA3* and *RPL3* (Figure 3H). Hence, *RBP95* is part of the *NPA1* genetic network, suggesting that it likely functions together with the Npa1 complex and Rpl3 in early pre-60S maturation.

### *RBP95* deletion enhances the pre-rRNA processing defects of *dbp6* and *dbp9* mutants

**Figure 4.**
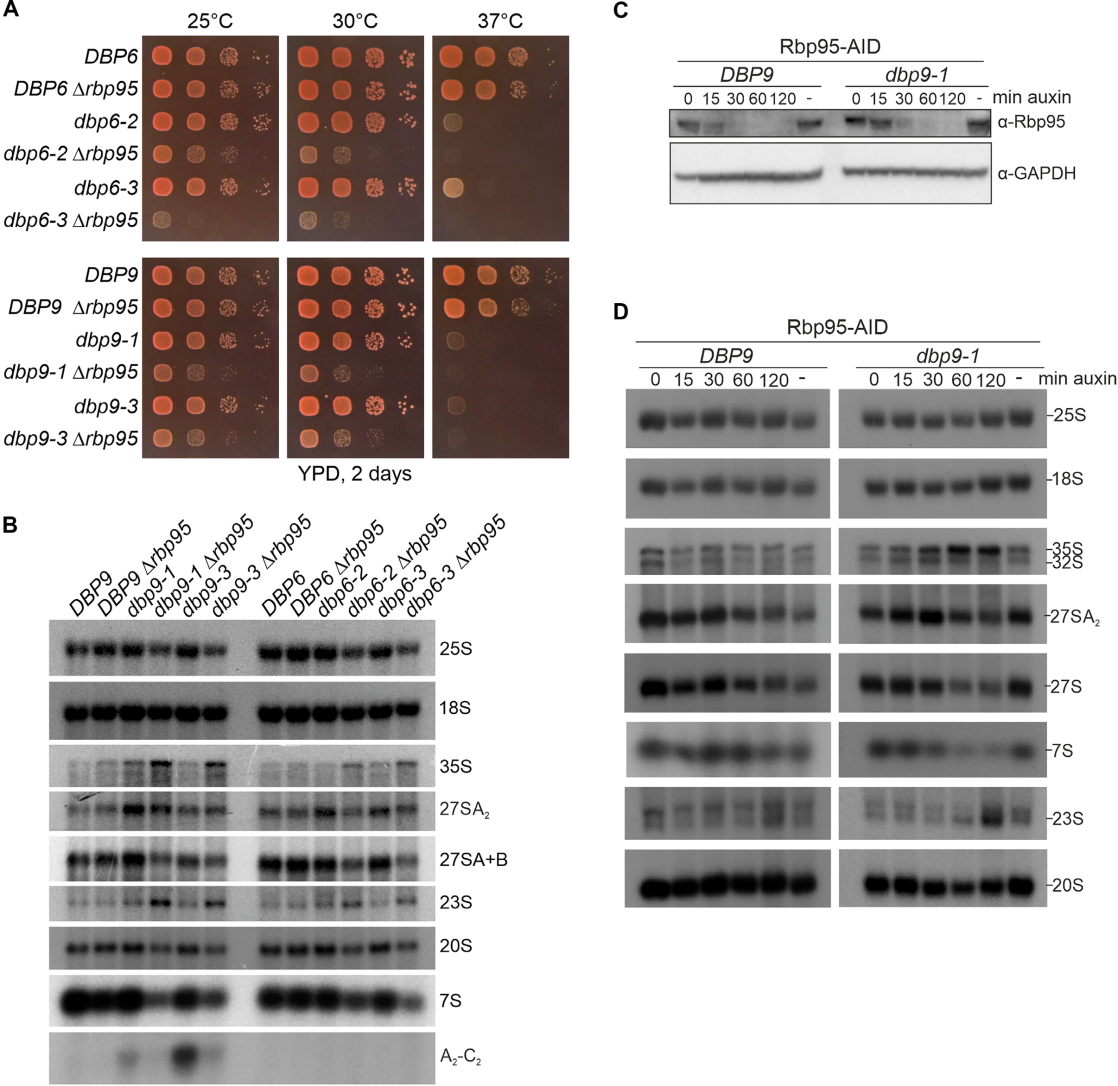
*RBP95* deletion enhances the pre-rRNA processing defects of *dbp6* and *dbp9* mutants. (**A**) Genetic enhancement of growth defects of *dbp6* and *dbp9* mutants upon deletion of *RBP95*. Single Δ*dbp6* and Δ*dbp9* as well as double Δ*dbp6* Δ*rbp95* and Δ*dbp9* Δ*rbp95* mutant cells, harboring plasmids containing either wild-type *DBP6* and *DBP9* or the indicated *dbp6* and *dbp9* mutant alleles (recovered from the 5-FOA-containing plates shown in Supplementary Figure S3), were spotted in serial dilutions on YPD plates, which were incubated for 2 days at the indicated temperatures. (**B**) RNA was extracted from cells with the indicated genotypes that had been grown at 30°C. Pre-rRNA processing intermediates were detected by northern blotting using probes 25S, 18S, A2-A3 (27SA_2_, 35S, and 23S pre-rRNAs and A_2_-C_2_ fragment), E-C2 (27SA, 27SB, and 7S pre-rRNAs), and D-A2 (20S pre-rRNA). Quantifications of 35S, 27SA_2_, and 27SA + B signals are provided in Supplementary Figure S4A. (**C**) *DBP9* wild-type or *dbp9-1* mutant cells carrying additionally an Rbp95-AID tag fusion were incubated with 500 μM auxin (dissolved in ethanol) for the indicated time periods or with ethanol alone (–). The decrease of Rbp95 was followed by western blotting. GAPDH was used as a loading control. (**D**) RNA was extracted from cells grown as in (C) and northern blotting was performed with the same probes as in (B). Quantifications of 35S, 27SA_2_, and 27SA + B signals are provided in Supplementary Figure S4B.

To further confirm some of the genetic interactions uncovered in the screen, we performed Δ*rbp95* knockouts in *RPL3, DBP6* and *DBP9* shuffle strains and transformed the resulting strains with plasmids containing previously described *rpl3, dbp6* and *dbp9* alleles (16,48,50). The viability of the resulting strains was examined on 5-FOA-containing plates after shuffling out the complementing *DBP6*, *DBP9* and *RPL3* plasmids bearing the *URA3* marker (Supplementary Figure S3). These analyses indicated that *rpl3-101* Δ*rbp95* and *rpl3-102* Δ*rbp95* double mutants were inviable and hence showed synthetic lethality (Supplementary Figure S3A). Furthermore, while one of the tested *dbp6* mutants (*dbp6-4*) and one of the tested *dbp9* mutants (*dbp9-5*) were lethal in combination with Δ*rbp95*, two *dbp6* mutants (*dbp6-2* and *dbp6-3*) and two *dbp9* mutants (*dbp9-1* and *dbp9-3*) were viable in combination with Δ*rbp95*, although their growth was substantially affected (Supplementary Figure S3B, C). Next, we assessed the growth phenotypes of these viable mutants at different temperatures (Figure 4A). While all tested *dbp6* and *dbp9* single mutants were thermosensitive and showed very poor or no growth at 37°C, they grew almost like wild-type cells at 25 and 30°C. However, these mutants displayed severe growth defects in combination with Δ*rbp95*, especially at 30°C, indicating a strong genetic interaction at this temperature (Figure 4A).

Based on these growth phenotypes, we reasoned that 30°C would be the ideal growth temperature to further investigate if *RBP95* deletion aggravates ribosome biogenesis phenotypes of the otherwise mild *dbp6* and *dbp9* single mutants. At this temperature the *dbp6* and *dbp9* mutants only showed growth defects when *RBP95* was additionally deleted.

We extracted RNA from these mutants and detected intermediates of the pre-rRNA processing pathway by northern blotting (Figure 4B, Supplementary Figure S4A). The *dbp9-1*, *dbp6-2*, and *dbp6-3* mutants, despite showing no or only subtle growth defects (Figure 4A), slightly accumulated the 27SA_2_ pre-rRNA (Figure 4B). In contrast to that, the 35S and 23S pre-rRNAs, but not 27SA_2_ pre-rRNA, accumulated in the tested *dbp6* Δ*rbp95* and *dbp9* Δ*rbp95* double mutants, which is indicative of early pre-rRNA processing defects at sites A_0_, A_1_ and A_2_. Probably as a result, less 27S pre-rRNAs and, consequently, less 7S pre-rRNAs and less mature 25S rRNAs were formed. Additionally, the blockage at early steps also led to reduced amounts of the 20S pre-rRNA, although almost normal levels of mature 18S rRNA were generated. Notably, these pre-rRNA processing phenotypes strongly resemble the phenotypes of Dbp6 and Dbp9 depletion (48,51). Hence, the deletion of *RBP95* in mild *dbp6* and *dbp9* mutants phenocopies the effect of Dbp6 and Dbp9 depletion. Besides that, we noticed that in *dbp9* but not in *dbp6* mutants, an A_2_-C_2_ fragment was detectable (Figure 4B), previously interpreted as an indication for pre-mature C_2_ cleavage (52). The signal of this fragment decreased when *RBP95* was additionally deleted, suggesting that less amount of this fragment accumulated due to the blockage at earlier steps.

As 35S and 23S pre-rRNA frequently accumulate as a secondary consequence of other processing defects, we sought to obtain a more kinetic picture of the chronology of pre-rRNA processing defects arising as a consequence of Rbp95 depletion. For this purpose, we established a system in which Rbp95 was fused to an auxin-inducible degron (AID) tag, allowing its quick degradation upon addition of auxin. We used this system to degrade Rbp95 in a *DBP9* wild-type or in the *dbp9-1* mutant strain. Indeed, Rbp95 levels were already substantially reduced after 15 min of auxin treatment and Rbp95 was almost undetectable after 30 min of auxin treatment in both strains (Figure 4C). We then extracted RNA from cells after auxin-induced Rbp95 degradation and analyzed pre-rRNA processing by northern blotting (Figure 4D, Supplementary Figure S4B). The first observable effect upon Rbp95 degradation in the *dbp9-1* mutant was 27SA_2_ pre-rRNA accumulation, which was apparent already after 15 min and 30 min of auxin treatment, before the signal decreased again. 35S pre-rRNA accumulated as well, but reached maximal levels only after 60 min of auxin treatment. The delayed accumulation of this precursor might indicate that 35S accumulation is actually a feedback effect of inhibition of 27SA_2_ processing. As the high levels of 35S pre-rRNA observed after 60 and 120 min of auxin incubation coincided with a substantial reduction of the 27SA_2_ precursor, we presume that 27SA_2_ reduction is the consequence of 35S accumulation. Additionally, part of the 27SA_2_ reduction might be the consequence of partial degradation of faulty 27SA_2_ containing pre-60S particles. Last but not least, increased levels of 23S pre-rRNA were observed after 120 min. To conclude, our data suggest that the main defect of *dbp9-1* mutants lacking Rbp95 is in processing of the 27SA_2_ pre-rRNA, suggesting that Rbp95 and the Npa1 complex cooperate during early pre-60S maturation.

### Rbp95 binds to helix H95 in domain VI of the 25S rRNA

The observed genetic interactions (Figures 3, 4, and Supplementary Figure S3) as well as the co-enrichment of Npa1 complex members with Rbp95-TAP (Figure 2D) suggest that Rbp95 might be in close proximity or even in direct physical contact with components of the Npa1 complex. However, no physical interactions between Rbp95 and any of the Npa1 complex members were observed in Y2H assays (data not shown).

According to the yeast genome database (www.yeastgenome.org), Rbp95 has a likely human ortholog, C7orf50 (see also alignment in Supplementary Figure S7A), which was reported to be an RNA-binding protein (53). C7orf50 carries an uncharacterized domain, DUF2373, shared with Rbp95, recently proposed to be renamed WKF domain (indicated in blue in Supplementary Figure S7A). This domain was shown to interact with RNA (54). Based on this, we speculated that the primary binding partner of Rbp95 might not be a protein but a (pre-)rRNA segment.

**Figure 5.**
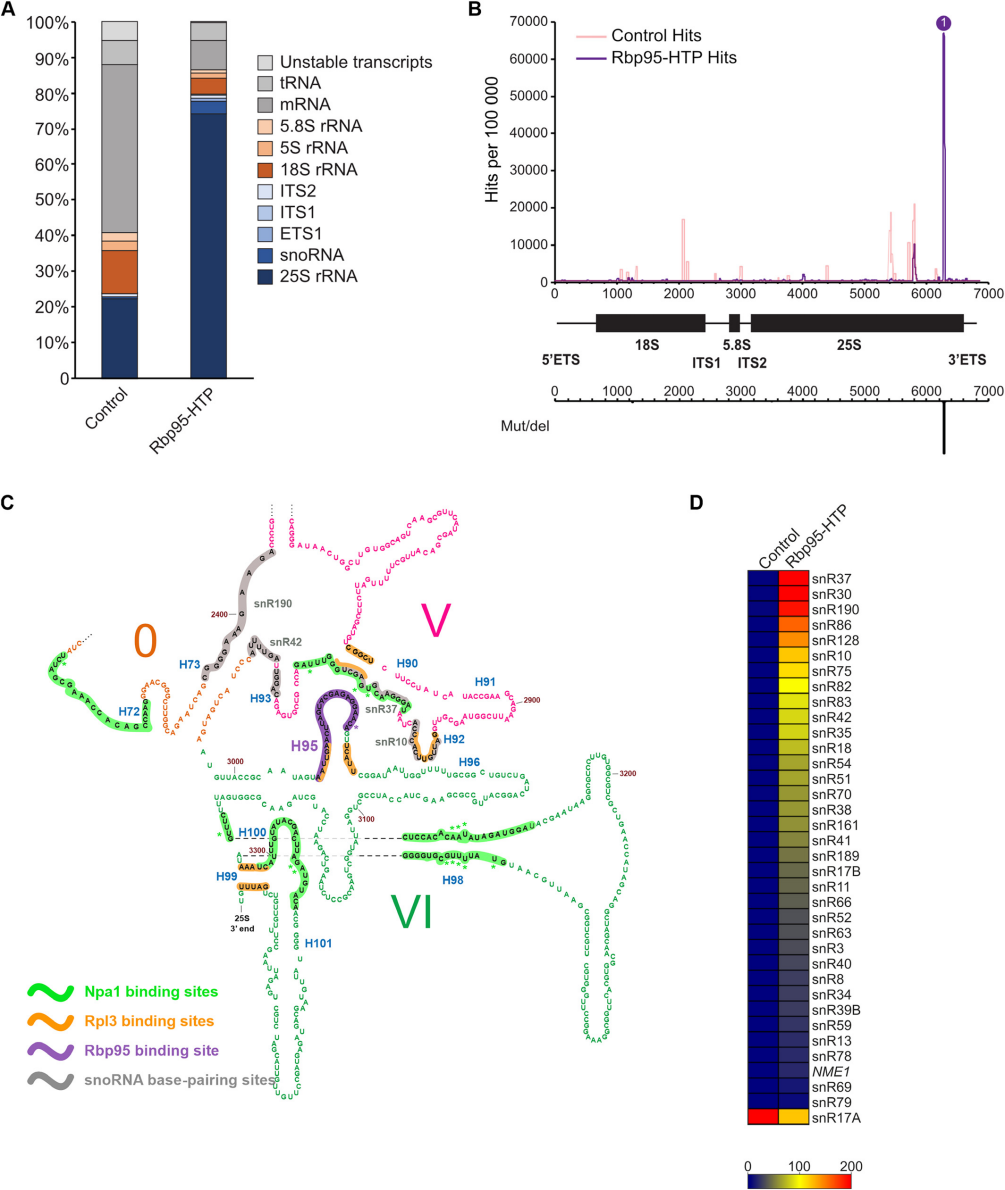
CRAC analyses identify a Rbp95-binding site in helix H95 in domain VI of the 25S rRNA. Rbp95-HTP-expressing cells and control cells (BY4741 wild-type cells) were subjected to UV-crosslinking, the RNAs bound to Rbp95 were purified, reverse transcribed, and the cDNAs were sequenced (for details, see Materials and Methods). (**A**) The relative distribution of sequences corresponding to the indicated RNA types is shown, revealing an enrichment of sequences of the 25S rRNA and snoRNAs compared to the negative control. (**B**) The obtained sequences were aligned with the 35S pre-rRNA and the number of hits for each nucleotide was plotted, revealing a prominent peak at the 3′-end of the 25S rRNA associated, as indicated below, with a peak of mutations/deletions. All identified mutations and deletions map to C3034, suggesting that this nucleotide corresponds to the actual Rbp95 crosslinking site. (**C**) Section of the 25S rRNA secondary structure (http://apollo.chemistry.gatech.edu/RiboVision/#SC_LSU_3D, (73)), including domain VI and parts of domains V and 0. The Rbp95-binding site within helix H95 is highlighted in violet, and the violet asterisk indicates the crosslinked nucleotide C3034. In addition, the previously identified Npa1-binding sites are highlighted in green, and crosslinked nucleotides therein are indicated by green asterisks (10). The Rpl3-binding sites, as observed in the X-ray structure of the mature 60S subunit (74), are shown in orange. Base-pairing regions of enriched snoRNAs in proximity of H95 (snR37, snR190, snR10, snR42) are highlighted in gray. See Supplementary Figure S5 for the full 25S rRNA secondary structure. (**D**) Identified snoRNAs in the Rbp95-CRAC experiment and a negative control purification from wild-type strain BY4741. The heat map reflects the number of reads per 100 000 total reads; snoRNAs with 200 or more reads per 100 000 total reads are shown in red and snoRNAs with less reads are represented as indicated in the grading scale at the bottom. The actual proportion of reads for each snoRNA is presented in Supplementary Table S5.

To find out whether Rbp95 interacts with rRNA and identify its binding site, we performed RNA crosslinking and analysis of cDNA (CRAC) analyses (55). Wild-type yeast or a strain expressing Rbp95 fused to a C-terminal His6-TEV-proteinA (HTP) tag was subjected to *in vivo* crosslinking and Rbp95 and its associated proteins and RNAs were subsequently purified and partially digested with RNases. RNA fragments bound to Rbp95 were then isolated, reverse transcribed, PCR-amplified, and the resulting cDNA library was subjected to deep sequencing. The obtained sequences were mapped to the yeast genome and the proportions of reads derived from the different RNA families were analyzed. According to these results, the majority of reads (more than 70%) obtained with Rbp95-HTP originated from the 25S rRNA (Figure 5A). Mapping of the distribution of reads in the sequence of the 35S pre-rRNA indicated one single prominent peak close to the 3′-end of the 25S rRNA (Figure 5B). The crosslinked sequences lie within helix H95 in domain VI of the 25S rRNA (Figure 5C). Due to the strong and specific crosslinking to this site, we propose to rename Ycr016w into Rbp95 (rRNA-binding protein helix H95). A large number of sequence reads of helix H95 carried deletions or substitutions of C3034, suggesting that this residue is the actual site of crosslinking. Interestingly, this site is in immediate proximity to the binding site of Rpl3 within helix H95 (Figure 5C, Supplementary Figure S5). Additionally, the crosslinking sites of Npa1 within domain VI of the 25S rRNA are also close to the Rbp95 binding site (Figure 5C). These results further corroborate that Rbp95 and the Npa1 complex as well as Rpl3 act together in early pre-60S maturation.

Beside the 25S rRNA, a second RNA type was enriched in the Rbp95-HTP CRAC analysis compared to the control, namely snoRNAs (Figure 5A). Notably, more than 30 different snoRNAs were crosslinked to Rbp95, and for most of them, also deletions/substitutions were identified in the corresponding cDNA sequences, further strengthening these data (Figure 5D, Supplementary Table S5). The highest represented snoRNA in the CRAC data was snR37, followed by snR30 and snR190. The H/ACA box snoRNP snR37 pseudouridylates U2944 in helix H92 of 25S rRNA domain V (Figure 5C (56)). The C/D box snoRNA snR190 presumably does not modify RNA but instead probably functions as an RNA chaperone that base-pairs to two different sites in early pre-60S particles, one of them in domain I and the second one in helix H73 of domain V (Figure 5C and (57)). Additionally, two other H/ACA box snoRNAs that were crosslinked to Rbp95, snR10 and snR42, bind to helix H92 and helix H93 in domain V and mediate pseudouridylation of U2923 and U2975, respectively (Figure 5C (56)). Although the relative positioning of domain V and VI is not known in early pre-60S particles, the above discussed snoRNA binding/modification sites are in proximity to helix H95 in later pre-60S particles ((58,59), Supplementary Figure S6), suggesting that Rbp95 might be contacting helix H95 and these snoRNAs at the same time. In contrast, the second most enriched snoRNA, snR30, has no known binding site in the 25S rRNA, but is instead known to base-pair with 18S rRNA and to be essential for early pre-rRNA processing steps (14). The presence of snR30 among the Rbp95 binding targets is consistent with the observed co-purification of the 35S and 23S pre-rRNAs with Rbp95-TAP (Figure 2B).

In addition to snR30 and the snoRNAs binding to 25S rRNA domain V, many other snoRNA sequences were also detected in the CRAC experiment, although at lower levels. The reason for the crosslinking of these snoRNAs is not clear at this point; however, it has to be considered that Rbp95 may, in addition to its role in pre-60S particle maturation, have also a snoRNA-related function.

### Rbp95 is a general RNA-binding protein

**Figure 6.**
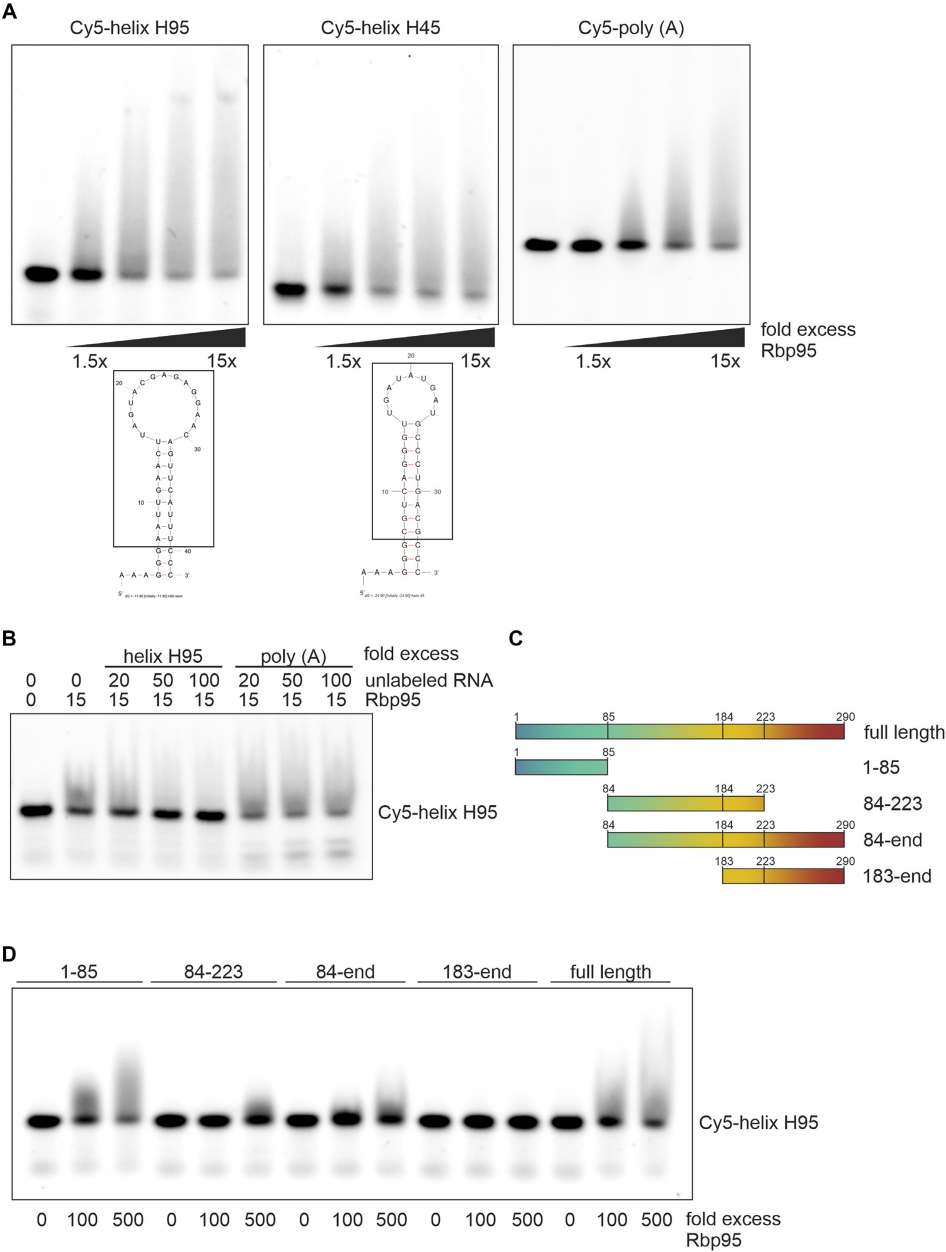
Rbp95 is an RNA-binding protein. (**A**) Cy5-labeled RNA oligonucleotides comprising the sequences of 25S rRNA helices H95, H45 or the Cy5-labeled poly(A) RNA oligonucleotide were incubated with increasing molar excess (1.5 to 15-fold) of purified Rbp95. Then, samples were separated on 4–20% Novex TBE gels, and Cy5-signals were subsequently detected on a Bio-Rad ChemidocMP imaging system. The secondary structures of the RNA fragments, predicted by Mfold (75), are shown below. The part corresponding to the respective sequence in the 25S rRNA is indicated by a box. (**B**) Competition experiments. The Cy5-labeled H95 RNA oligonucleotide was incubated with a 10-fold excess of Rbp95 and, in addition, either a 0-, 20-, 50-, or 100-fold excess of unlabeled oligo H95 or poly(A) RNA oligonucleotide relative to the labeled oligonucleotide. (**C**) Schematic overview of the used, truncated Rbp95 fragments. (**D**) Cy5-labeled RNA H95 oligonucleotide was incubated without, or with 100- or 500-fold molar excess of the indicated purified Rbp95 fragments.

Given the very specific crosslinking of Rbp95 to helix H95 of the 25S rRNA, we wondered whether Rbp95 is an RNA-binding protein with specificity for the sequence or structure of helix H95. To address this, we purified recombinant Rbp95 and performed electrophoretic mobility shift assays (EMSAs) with a 5′-Cy5-labeled RNA oligonucleotide comprising the sequence of helix H95, with the stem extended by three G-C pairs in order to stabilize its secondary structure and three unpaired As for 5′-attachement of Cy5 (Figure 6A). Incubation of Cy5-helix H95 with Rbp95 resulted in a strong shift of the Cy5-helix H95 band, indicating that Rbp95 binds to this RNA (Figure 6A, left panel). To test if Rbp95 could also recognize other helices with similar conformations, we also tested 5′-Cy5-labeled helix H45, another 25S rRNA helix that is not bound by Rbp95 *in vivo*. However, Rbp95 bound to this helix similarly well as to helix H95 (Figure 6A, middle panel). To find out whether Rbp95 is able to bind to single-stranded RNA as well, we used 5′-Cy5-labeled poly(A) RNA. We observed that Rbp95 also bound to single-stranded RNA. However, while the helix H45 and H95 bands already started to shift at 1.5-fold excess of Rbp95, an at least ∼4-fold excess of Rbp95 was necessary to shift Cy5-poly(A) (Figure 6A, right panel).

To further confirm that Rbp95 binds better to stem-loop RNA structures than to single-stranded poly(A) RNA, we performed competition assays (Figure 6B). While an excess of unlabeled helix H95 RNA was able to revert the shift of Cy5-labeled helix H95 RNA, competition by unlabeled poly(A) RNA was very ineffective even when added in high excess (Figure 6B).

Taken together, we conclude that Rbp95 is a non-specific RNA-binding protein with higher affinity for stem-loop structures than for single-stranded RNA. As Rbp95 binds to 25S RNA helix H95 very specifically *in vivo*, we infer that, beside Rbp95 and the target RNA, other determinants probably ensure this specific binding *in vivo*.

The observation that Rbp95 not only crosslinks to helix H95 but also to snoRNAs raises the idea that Rbp95 might bind to helix H95 and a snoRNA at the same time. Presumably, such dual binding of different RNAs would require Rbp95 to harbor more than one RNA-binding domain. To further follow this hypothesis, we investigated which Rbp95 subdomains are capable of binding to RNA (Figure 6C, Supplementary Figure S7B).

As C7orf50′s WKF domain is sufficient for interaction with RNA (54), we aimed at testing the corresponding yeast WKF domain for its capability to bind to RNA. According to structural predictions, amino acid 119, which would correspond to the start of the WKF domain in Rbp95, lies within an α-helix (Supplementary Figure S7B). In order to preserve this α-helix in our experiments, we decided to test an extended WKF domain also including this α-helix lying N-terminal to the WKF domain (84-end fragment). Complementary to that, we tested the N-terminal fragment until amino acid 85 (1–85). Moreover, as the C-terminal part of Rbp95 from amino acid 223 onwards is predicted to be mostly unstructured, we also tested a sub-fragment of the WKF domain lacking this sequence (84–223 fragment). Last but not least, we tested the C-terminal fragment from amino acid 183 onwards.

All truncated Rbp95 fragments and full-length Rbp95 were purified (Supplementary Figure S7C) and tested for binding to the helix H95 RNA.

Strikingly, the Rbp95 1–85 fragment, not harboring any so far known RNA-binding domain, induced a strong migration shift of helix H95 RNA (Figure 6D), which required however slightly higher protein concentrations than the shift caused by full-length Rbp95 (Supplementary Figure S7D). Also, the C-terminal (WKF) fragment from amino acid 84 onwards (84-end) bound to helix H95 RNA, albeit much weaker than the 1–85 fragment (Figure 6D and Supplementary Figure S7D). The WKF domain was also able to bind to helix H95 RNA when the unstructured part from amino acid 223 onwards was missing (84–223), however, to a reduced extent, suggesting that also the very C-terminal part contributes to fully efficient binding of the WKF fragment (Figure 6D). In contrast, the C-terminal part from amino acid 183 onwards alone was not sufficient for RNA binding (Figure 6D). In summary, we conclude that Rbp95 contains two RNA-binding domains, an N-terminal one with higher affinity and a central one with contribution of the C-terminal part with lower affinity. The maximal *in vitro* RNA binding activity was observed with the full-length protein containing both domains.

### TurboID proximity labeling reveals 90S and pre-60S factors in physical proximity to Rbp95

**Figure 7.**
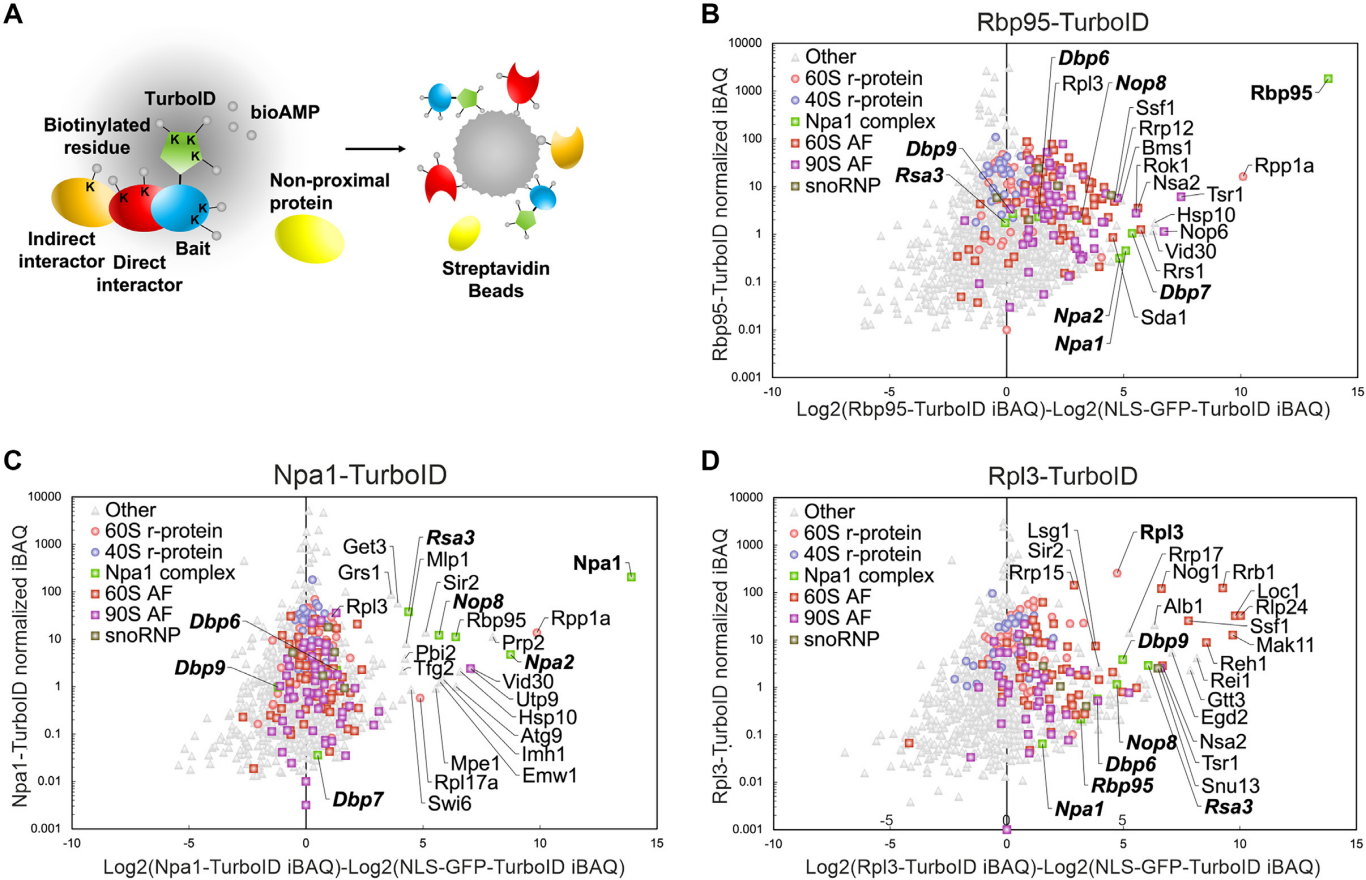
TurboID indicates physical proximity of Rbp95 to 90S and pre-60S AFs. (**A**) Schematic overview of the TurboID method. The bait protein (blue) is fused at its C-terminus to the TurboID biotin ligase, which efficiently enables, by catalyzing the conversion of biotin into the reactive biotinoyl-5′-AMP intermediate (bioAMP), the covalent biotinylation of lysine (K) residues of proteins that are in close physical proximity to the enzyme, such as the bait protein as well as its direct (red) or indirect (orange) interaction partners. Biotinylated proteins are then purified via streptavidin beads and subsequently identified by mass spectrometry. (**B–D**) TurboID results with Rbp95 (B), Npa1 (C), and Rpl3 (D) as baits. The normalized abundance value (iBAQ, intensity-based absolute quantification) of each protein detected in the respective purification is plotted against its relative abundance (log_2_-transformed enrichment) compared to the abundance in the control purification from cells expressing the NLS-GFP-TurboID bait. Hence, proteins that are enriched compared to the negative control can be found on the right side of the Christmas tree plot. The names of proteins that are particularly enriched are indicated. The bait proteins are highlighted by bold letters, and Rbp95 as well as all Npa1 network members are written in bold, italic letters.

To find out if proteins positioned close to helix H95 or interacting with snoRNAs can be detected in physical proximity to Rbp95, we performed TurboID-based proximity labeling (Figure 7A). This method is based on the fusion of a bait protein to TurboID, an improved variant of the promiscuous biotin ligase BirA* (also referred to as BioID), which efficiently biotinylates proteins that are in close physical proximity to the enzyme (60–63). Biotinylated proteins are then affinity-purified via streptavidin beads and identified by mass spectrometry. Although TurboID using Rbp95 as bait did not reveal any single protein to be substantially more co-enriched than all others (which would be expected for a direct interaction partner), Npa1 and its network members Npa2, Dbp7, and Nop8 were clearly enriched compared to the negative control (Figure 7B, Supplementary Table S6). Additionally, several other pre-60S AFs, such as Ssf1, Nsa2, Sda1 and Rrs1, and also the 90S factors Rok1, Bms1, Rrp12, and Nop6 were enriched (Figure 6B), suggesting that Rbp95 might be bound to pre-ribosomal particles in proximity to these proteins (see also Discussion). *Vice versa*, Rbp95 was strongly enriched in the Npa1 TurboID, besides the Npa1 complex members Npa2, Nop8 and Rsa3 (Figure 7C, Supplementary Table S6). Additionally, in the TurboID experiment with Rpl3, several Npa1 complex members as well as Rbp95 were among the enriched proteins (Figure 7D, Supplementary Table S6).

### Npa1 and Rpl3 are required for efficient Rbp95 binding

Considering the physical proximity of Npa1 and Rpl3 to Rbp95 revealed in the TurboID experiment, together with the proximal positioning of these proteins to helix H95 in pre-60S structures (Rpl3) or CRAC analyses (Npa1), we next assessed whether these proteins are required for recruitment of Rbp95 to pre-60S particles. Additionally, we investigated if Ssf1 could play a role in Rbp95 recruitment, based on its enrichment in the Rbp95- and Rpl3-TurboID experiments and the fact that Ssf1 is positioned in pre-60S particles close to Rpl3 and Npa1 (Figure 9). To address this, we used Noc2-TAP strains in which *RPL3*, *NPA1*, and *SSF1*, respectively, were under the control of the glucose-repressible *GAL1* promoter. To prevent that its paralog Ssf2 takes over Ssf1′s function when the latter is depleted, we additionally deleted *SSF2* in the *GAL1*-*SSF1* strain. Surprisingly, shifting the wild-type control strain from galactose to glucose containing medium resulted in a substantial increase of Rbp95 co-purification with Noc2-TAP (Supplementary Figure S8A). As also substantially higher amounts of Rbp95 were detected in the lysates of cells grown in glucose-containing medium compared to galactose-containing medium (Supplementary Figure S8B), Rbp95 seems to be expressed at much higher levels in the presence of glucose as carbon source compared to galactose.

Compared to the wild-type situation, Rpl3 or Npa1 depletion, and to a lesser extent Ssf1 depletion, resulted in a substantial reduction of Rbp95 levels bound to Noc2-TAP particles (compare Rbp95 levels in the glucose conditions; Supplementary Figure S8A). However, the Ssf1 reduction was not very prominent anymore when considering that also in the lysates, Rbp95 levels were lower in glucose conditions than in the wild-type (see ratios of Rbp95 levels in TEV eluates relative to lysates in Supplementary Figure S8C). The reduced levels of Rbp95 bound to Noc2-TAP particles in the Rpl3-, Npa1- and potentially to some extent also in Ssf1- depleted strains compared to the wild-type control suggests reduced recruitment of Rbp95 in these conditions. We conclude that in the absence of Npa1, Rpl3, or Ssf1, Rbp95 binding to early pre-60S particles is hampered. Additionally, in line with previous results (10), Npa1 depletion also resulted in a severe reduction of pre-60S-bound Dbp6. Moreover, also pre-60S bound Rpl3 was reduced upon Npa1 depletion. Conversely, Rpl3 depletion also resulted in a slight reduction of pre-60S-bound Npa1 and Dbp6 (Supplementary Figure S8A). We conclude that Npa1 or Rpl3 depletion has a severe impact on the composition of early pre-60S particles, including the reduction of Npa1 complex members and Rbp95.

### *RBP95* deletion alters the composition of early pre-60S particles

**Figure 8.**
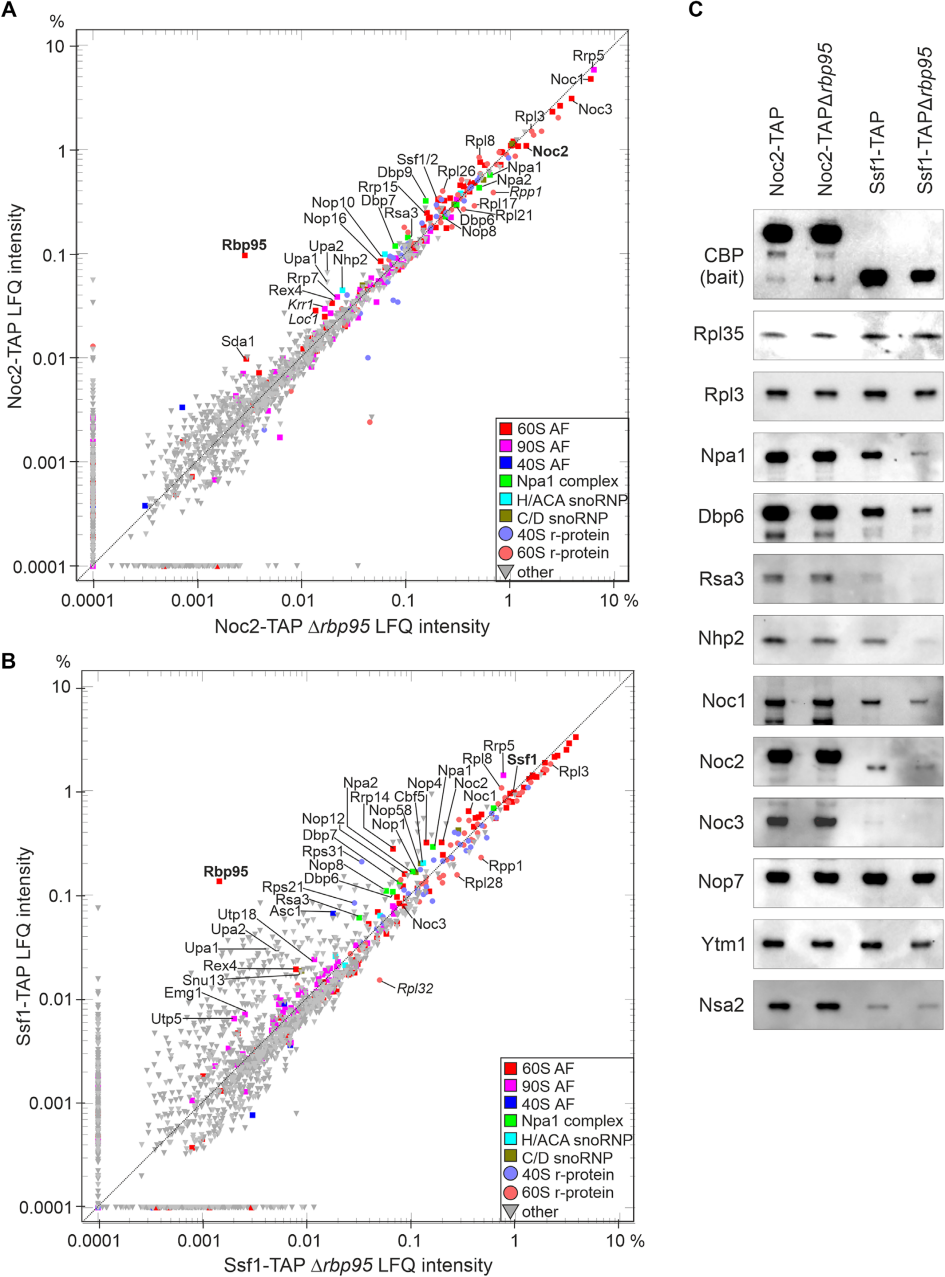
The absence of Rbp95 alters the composition of early pre-60S particles. (**A, B**) Comparison of proteins co-purified with Noc2-TAP (A) and Ssf1-TAP (B) in the presence or absence of Rbp95 by label-free semi-quantitative mass spectrometry. LFQ intensity values are indicated in percent relative to the total intensity of proteins detected in the respective purification. Proteins particularly enriched in one of the purifications as well as Noc1, Noc2, Noc3, Rrp5, Rpl3, and Npa1 complex members are labeled, and bait proteins and Rbp95 are indicated in bold letters. The relative signal intensities represent the means of two biological replicates. Proteins that were enriched/decreased in only one of the two experiments (and should therefore not be considered further) are indicated in italic letters. (**C**) Eluates of TAP purifications as in (A) and (B) were analyzed by western blotting using antibodies recognizing the H/ACA-box snoRNP component Nhp2, the indicated AFs, and the large subunit r-proteins Rpl35 and Rpl3.

A potential function of Rbp95 in early pre-60S particles might be to promote the recruitment of assembly factors either directly or by facilitating rRNA folding steps that consequently favor the recruitment of proteins. To experimentally address this hypothesis, we purified early pre-60S particles from wild-type and Δ*rbp95* strains and compared their protein composition by label-free semi-quantitative mass spectrometry (Figure 8A and B) and by western blotting (Figure 8C). As we had observed high levels of Rbp95 in pre-60S particles purified via Noc2-TAP and Ssf1-TAP (Figure 2B), we selected these particles for this analysis.

As a rather broad range of early pre-60S particles co-purifies with Noc2-TAP, alterations in the dynamics of early maturation steps should alter the overall composition of these particles (i.e. a shift in the distribution of earlier versus later particles). Such overall changes in Noc2-TAP particle composition due to *RBP95* deletion were however not apparent, as indicated by the fact that the relative levels of co-purification of Noc1 and Noc3, characteristic of the earlier and later subpopulation of Noc2-TAP particles, respectively, did not change in the *Δrbp95* mutant (Figure 8A, C, Supplementary Table S4). Also, despite the fact that Rpl3 and Npa1 bind pre-60S particles in proximity to Rbp95, and the observation that their depletion prevents Rbp95 binding to pre-60S particles (Supplementary Figure S8), neither Rpl3 nor Npa1 levels changed in Noc2-TAP particles, indicating that the presence or absence of Rbp95 bound to helix H95 does not influence Rpl3 or Npa1 binding (Figure 8A, C). Interestingly, the levels of only few proteins were reduced, suggesting that absence of Rbp95 does not delay overall maturation of Noc2-TAP particles, but rather has local effects at specific sites in pre-60S particles. The levels of Dbp7 and Dbp9, both part of the Npa1 genetic network (however not part of the so far described Npa1 physical complex), were reduced in the Noc2-TAP particles in the absence of Rbp95 (Figure 8A). Additionally, a reduction of H/ACA snoRNP components (in particular Nhp2 (Figure 8A and C) and Nop10 (Figure 8A)) was observed. Remarkably, the pre-60S factor Ssf1, which binds to 25S rRNA domain VI and is suggested to be in proximity to Rbp95 according to the TurboID experiment (Figure 7), and its paralog Ssf2 were reduced in the absence of Rbp95 (Figure 8A). Rrp15, a direct binding partner of Ssf1, was also reduced. Also Sda1, a protein binding mainly to domain V and additionally forming contacts with domains II and IV (64), which was as well detected in proximity to Rbp95 in the TurboID experiment, was reduced (Figure 8A).

Additionally, a group of proteins not bound in proximity to Rbp95/helix H95 in the structures resolved so far, namely r-protein Rpl8, its binding partner Nop16, and Rex4, a putative exonuclease with so far unknown function (65), were also found to be reduced in the Noc2-TAP particles from the Δ*rbp95* strain (Figure 8A). Only one 90S factor, Rrp7, was clearly reduced in this experiment. Last but not least, Upa1 and Upa2, the two novel pre-60S factors found to be co-enriched with Rbp95-TAP (Figure 2D), were strongly reduced in the absence of Rbp95, suggesting their stable binding relies on the presence of Rbp95.

The reduction of Ssf1 in the Noc2-TAP particles in the absence of Rbp95 suggested that Ssf1 might be less efficiently recruited to pre-60S particles. To gain a better understanding of the particles containing Ssf1 in the absence of Rbp95, we analyzed Ssf1-TAP particles from Δ*rbp95* cells. Interestingly, most AFs characteristic of very early pre-60S particles were reduced, including Rrp5, Noc1, Nop4, H/ACA as well as C/D box snoRNP proteins, and most Npa1 complex members (Figure 8B, C, Supplementary Table S4). Additionally, also the 90S factors Utp5, Utp18 and Emg1/Nep1, which were co-purified at low levels with Ssf1-TAP, were reduced in this purification. One of the most strongly reduced proteins was Rrp14, an interaction partner of Ssf1 within pre-60S particles (59). Similar to the Noc2-TAP experiment, the r-protein Rpl8, the putative exonuclease Rex4, and Upa1 and Upa2 were reduced in this purification, further confirming the less efficient recruitment of these factors in the absence of Rbp95 (Figure 8B). Hence, as mainly early pre-60S factors were reduced in the Ssf1-TAP purification in the absence of Rbp95, we hypothesize that Ssf1 is less stably bound to earlier pre-60S particles in the absence of Rbp95 (see also Discussion).

## DISCUSSION

In this study, we introduce a novel AF, Rbp95, which is predominantly bound to early, 27SA_2_ pre-rRNA-containing pre-60S particles. Genetic interaction with Rpl3 and several Npa1 complex members reveals a strong functional connection to the Npa1 complex.

**Figure 9.**
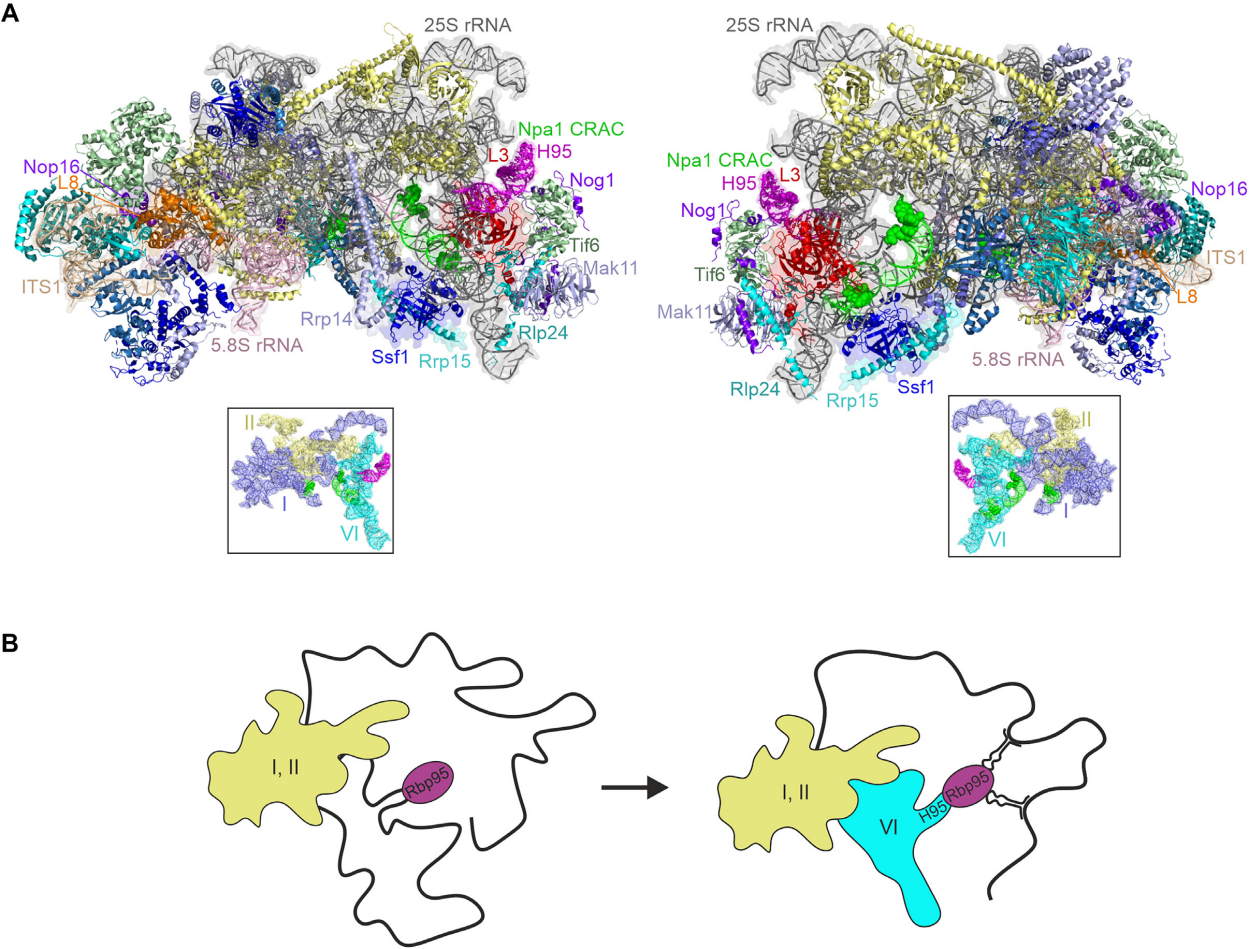
Model of Rbp95 binding and function. (**A**) Structural representation of an early pre-60S particle (State 2, PDB 6C0F) (59) Two different orientations (∼180° rotated) are shown. Helix H95 and the Npa1-binding sites are indicated in magenta and green, respectively, and nucleotides therein that are crosslinked to Rbp95 or Npa1 are shown in sphere representation. R-proteins are indicated in shades of yellow and AFs in shades of blue. Proteins in proximity to helix H95 and proteins discussed in this study are labeled. The boxes below show the three 25S rRNA domains that are visible at this maturation stage in different colors, with helix H95 labeled in magenta and the Npa1-binding sites in green. (**B**) Model for Rbp95 function. Rbp95 binds to helix H95 at a maturation stage when domains I and II (yellow) are folded, while domain VI (blue) is not. Rbp95 (violet) might, together with the Npa1 complex, participate in folding this domain. Moreover, Rbp95 binds to several snoRNAs base-pairing with domain V. By binding to domain VI and docking to domain V via the snoRNAs, Rbp95 may help to bring relevant rRNA elements into physical proximity for further folding and protein binding events.

Several proteins are in physical proximity to Rbp95 in pre-60S particles according to our TurboID experiments, including Rpl3, Npa1 and additional Npa1 network members. This finding is also supported by previous CRAC analyses, unraveling Npa1-binding sites in 25S rRNA domain VI, which are in short distance to helix H95 (Figure 9A, (10)). Additionally, one of the rRNA-binding sites of Rpl3 lies in helix H95 of domain VI (Figures 5C and 9A). The absence or presence of Rbp95 does not affect Rpl3 binding to early pre-60S particles (Figure 8A–C), suggesting that Rbp95 neither prevents nor favors Rpl3 binding. In contrast, in the absence of Rpl3 or Npa1, levels of early pre-60S particle-bound Rbp95 are greatly decreased (Supplementary Figure S8), suggesting that Rpl3 and/or Npa1 likely bind before Rbp95 and are important for the recruitment of Rbp95.

In addition to Npa1 complex members, also Ssf1 is suggested to be in proximity to Rbp95 based on the TurboID experiment. Ssf1 binds to domain VI helices H96 and H101 and is positioned in proximity to helix H95 in the earliest structures in which domain VI is visible, further supporting proximity between Ssf1 and Rbp95 (State 2, (59), Figure 9A). Interestingly, also the absence of Ssf1 seems to reduce Rbp95 binding to early pre-60S particles, although to a lesser extent than the absence of Rpl3 or Npa1 (Supplementary Figure S8). Vice versa, the absence of Rbp95 results in a reduction of Ssf1 and its binding partners Rrp14 and Rrp15 within Noc2-TAP pre-60S particles (Figure 8A, B). We hypothesize that Rbp95 and Ssf1 and its partners mutually stabilize their binding to early pre-60S particles.

*SSF1* depletion also leads to 35S accumulation and reduced 27SA_2_ levels, but later species decrease even more (52). Hence, similar to *RBP95* deletion, the primary defect in the absence of Ssf1 might be in the maturation of 27SA_2_ into later species. Interestingly, Ssf1 depletion also leads to the accumulation of an A_2_ to C_2_ fragment, suggesting premature cleavage at site C_2_. As a similar fragment was also found in *dbp9* mutants (Figure 4B), also Dbp9 might, potentially together with Ssf1, prevent premature C_2_ cleavage. Notably, reduced levels of this fragment were detected in *dbp9* mutants additionally carrying the *RBP95* deletion, likely as a consequence of the reduced 27SA_2_ pre-rRNA steady-state levels in this mutant, resulting in a reduction of all later species. The potential interplay of Ssf1, Dbp9 and Rbp95 in preventing C_2_ cleavage might be an interesting aspect to study in more detail in the future.

Besides, Nsa2 and Sda1 were also enriched in the Rbp95 TurboID experiment. Although these two AFs are predominantly associated with later pre-60S particles, they are also present in the Rbp95-TAP purification (Supplementary Table S4). Nsa2 makes a direct contact with helix H95 (58,64) and Sda1 engages in multiple rRNA contacts (in domains V, II and IV) (64); some of these might be in proximity to Rbp95 within early pre-60S particles. Moreover, binding of Sda1 to Noc2-TAP pre-60S particles was reduced in the absence of Rbp95.

As domain VI is flexible and hence not fully folded in the earliest pre-60S particles (58,59), we speculate that, by binding to helix H95, Rbp95 might promote folding of flexible parts of domain VI, potentially in conjunction with Npa1, which physically links domains I and II with domain VI ((10) Figure 9B). This might in turn facilitate recruitment of proteins binding to domain VI, such as Ssf1 and its binding partners Rrp14 and Rrp15.

Importantly, Rbp95 contains two different RNA-binding domains, opening the possibility that Rbp95 could bind two different RNA elements at the same time, which might help to draw different RNA domains together. CRAC data suggest that Rbp95 binds to several snoRNAs base-pairing with domain V of the 25S rRNA (i.e. snR37, snR190, snR10, snR42), suggesting that, via its interactions with helix H95 and snoRNAs, Rbp95 might bring domains V and VI into proximity (Figure 9B), thereby promoting further rRNA folding and protein recruitment. Of note, Npa1 also crosslinks to snR190, snR42, snR10, and, to a lesser extent, snR37 (10), further supporting the idea that Npa1 and Rbp95 cooperate in rRNA folding events, and that not only the binding of rRNA elements but also of snoRNAs is important in this context.

The interaction of Rbp95 with snoRNAs base-pairing with domain V might be the reason why in the absence of Rbp95, the levels of Rpl8 and its binding partner Nop16 were reduced in pre-60S particles. Rpl8 has a globular domain mainly binding to domain I of the 25S rRNA, while its N-terminal domain, which is accommodated later, binds to domain V (66).

In addition to these well-known pre-60S factors, the absence of Rbp95 also reduced the pre-60S association of the poorly characterized exonuclease Rex4 (65) and the novel pre-60S factors Upa1 and Upa2. Hence, our work may have revealed additional important players, potentially acting in conjunction with Rbp95, in these early pre-60S maturation steps. Indeed, a recent publication revealed Rbp95 to be a stochiometric component of Upa1- or Upa2-purified particles (45).

Due to the observations that Rbp95 is mainly bound to early pre-60S particles and is extensively linked to the Npa1 complex, we focused our study on the function of Rbp95 in early pre-60S particles. Nevertheless, in the course of our investigations, we found several pieces of evidence pointing towards an additional potential function of Rbp95 within 90S particles. A minor population of Rbp95 is present in 90S particles, as evidenced by the co-purification of low amounts of the 35S and 23S pre-rRNAs (Figure 2B) and some 90S AFs (Figures 1B, C and 2D). In line with this, the TurboID analyses suggest a physical proximity between Rbp95 and 90S factors, including Rok1, Bms1, Rrp12, Nop6, and Dhr2. Apart from that, the second most enriched snoRNA in the Rbp95-CRAC analyses was snR30, which base-pairs with the 18S rRNA but has no known binding site in pre-60S particles. Of note, snR10 also base-pairs with the 18S rRNA and is required for early pre-rRNA processing steps (67) in addition to guiding pseudouridylation of U2923 in domain V of the 25S rRNA. Interestingly, CRAC analyses revealed binding of Rok1 to both snR30 and snR10, and Rok1 was proposed to release snR30 (68,69). An alternative model suggests that Rok1 promotes Rrp5 release, which in turn is necessary for snR30 release (70). Interestingly, Rrp5 also directly binds to snR10 and snR30 according to CRAC analyses (8). Additionally, snR30 is required for the stable 90S recruitment of Rrp7 (71), a 90S AF that was reduced in Noc2-TAP particles when Rbp95 was absent. Another interesting connection is that snR30 shares one of its binding sites in the 18S rRNA with Bms1. Hence, the physical links of Rbp95 to Rok1, Bms1, snR30 and snR10, revealed by our TurboID and CRAC analyses, may indicate that Rbp95 might bind to 90S particles via snR30 and snR10 and thereby get into physical proximity to Rok1 and Bms1. Binding of Rbp95 to 90S particles via these snoRNAs would provide a possible explanation why the 23S rRNA, which does not contain helix H95 nor any other segments of 25S rRNA, was co-purified in the Rbp95-TAP purification (Figure 2B).

The presumable human Rbp95 ortholog C7orf50 localizes to the nucleolus according to the ‘human genome atlas’ (54,72) and is predicted to be functionally connected to several ribosome AFs including MAK16, NOP14, PES1, KRR1 and SDA1 (string-db.org). The WKF domain of C7orf50 was shown to be sufficient for RNA binding; however, up to now, it was not investigated whether the N-terminal domain of the protein can bind to RNA as well. Our findings about the two RNA-binding domains of Rbp95 and about the functional environment of Rbp95 may serve as paradigm to better understand the role of C7orf50 in human ribosome biogenesis.

## DATA AVAILABILITY

The MS proteomics data have been deposited to the ProteomeXchange Consortium via the PRIDE (21) partner repository with the dataset identifier PXD030106. NGS analysis files of raw and processed data were deposited in the Gene Expression Omnibus database under the accession number GSE189589. TurboID data are provided in Supplementary Table S6.

## Supplementary Material

gkac724_Supplemental_Files
