# Supplementary material for: Rbp95 binds to 25S rRNA helix H95 and cooperates with the Npa1 complex during early pre-60S particle maturation
Source: Nucleic Acids Res. 2022 Aug 26;50(17):10053–77. doi: 10.1093/nar/gkac724 (PMC9508819; doi:10.1093/nar/gkac724)
Supplement: gkac724_Supplemental_Files [file gkac724_supplemental_files.zip › Bhutadaetal_SupplementaryData.pdf]

Supplementary Data

Rbp95 binds to 25S rRNA helix H95 and cooperates with the Npa1 complex during early pre-60S particle maturation

Priya Bhutada[1], Sébastien Favre[2], Mariam Jaafar[3,4], Jutta Hafner[1,5], Laura Liesinger[5,6], Stefan Unterweger[1], Karin Bischof[1], Barbara Darnhofer[5,6], Devanarayanan Siva Sankar[2], Gerald Rechberger[1,5], Raghida Abou Merhi[4], Simon Lebaron[3], Ruth Birner-Gruenberger[5,6,7], Dieter Kressler[2], Anthony K. Henras[3], Brigitte Pertschy[1,5]

[1] Institute of Molecular Biosciences, University of Graz, Humboldtstrasse 50, 8010 Graz, Austria.

[2] Unit of Biochemistry, Department of Biology, University of Fribourg, Chemin du Musée 10, 1700 Fribourg, Switzerland.

[3] Molecular, Cellular and Developmental Biology Unit (MCD), Centre de Biologie Intégrative (CBI), Université de Toulouse, CNRS, UPS, 31062, Toulouse, France.

[4] Genomic Stability and Biotherapy (GSBT) Laboratory, Faculty of Sciences, Rafik Hariri Campus, Lebanese University, Beirut, Lebanon.

[5] BioTechMed-Graz, Graz, Austria.

[6] Diagnostic and Research Institute of Pathology, Medical University of Graz, 8010 Graz, Austria

[7] Institute of Chemical Technologies and Analytics, Technische Universität Wien, Getreidemarkt 9/E164, 1060 Vienna, Austria.

Supplementary Figure 1

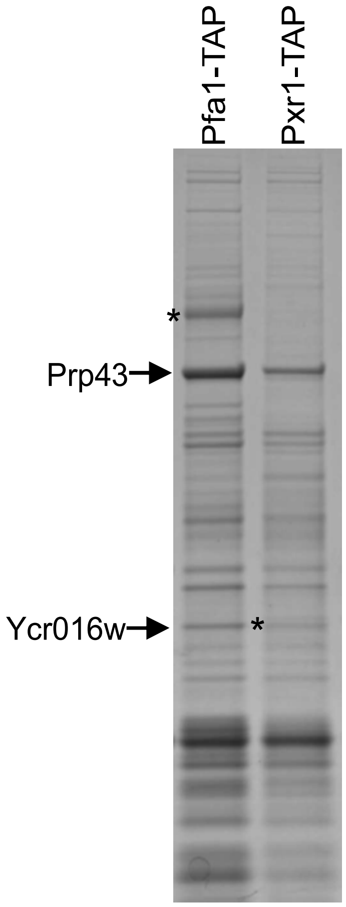

Supplementary Figure 2

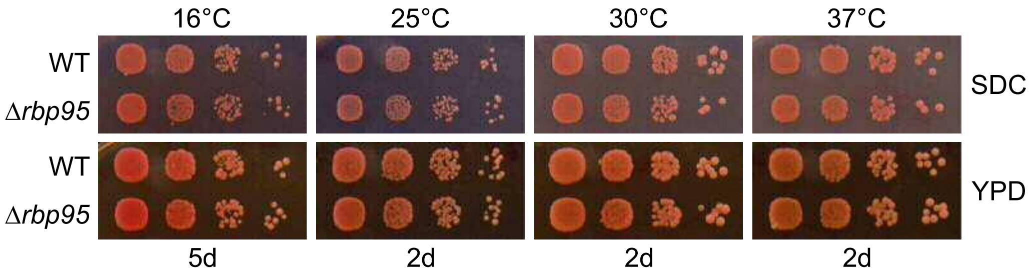

Supplementary Figure 3

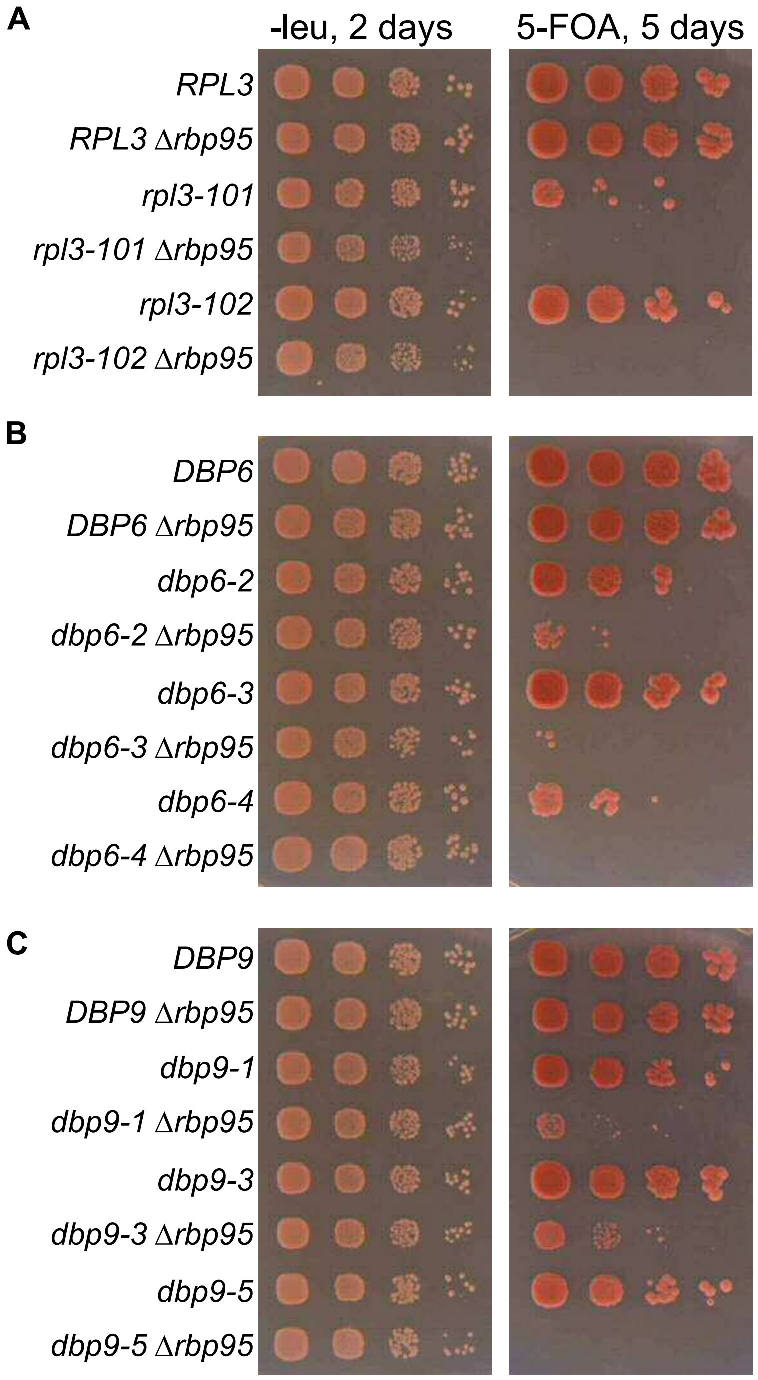

Supplementary Figure 4

A

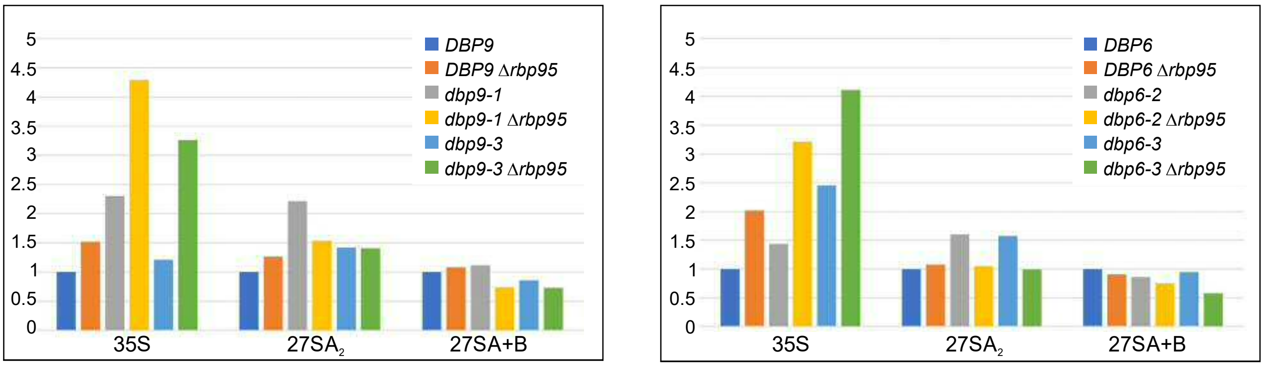

B

Rbp95-AID

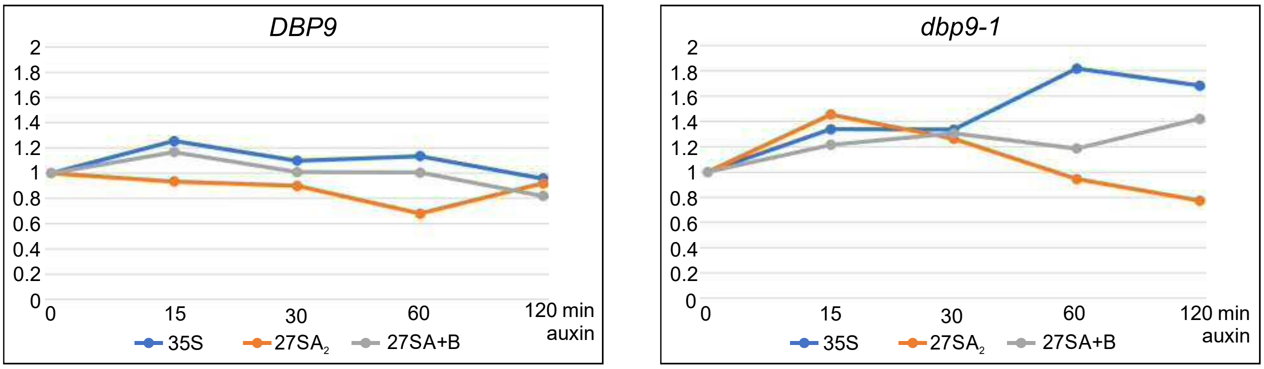

Supplementary Figure 5

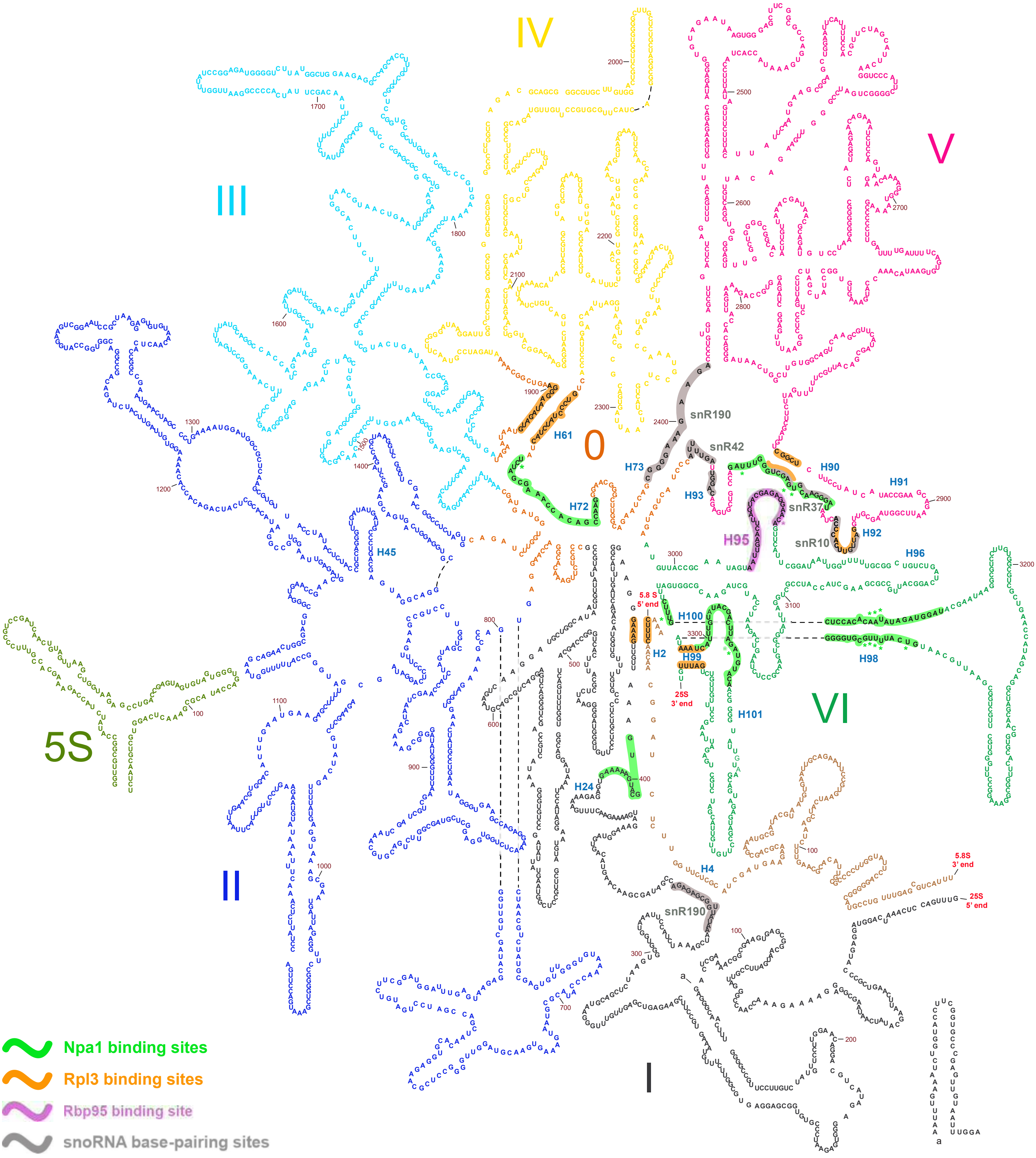

Supplementary Figure 6

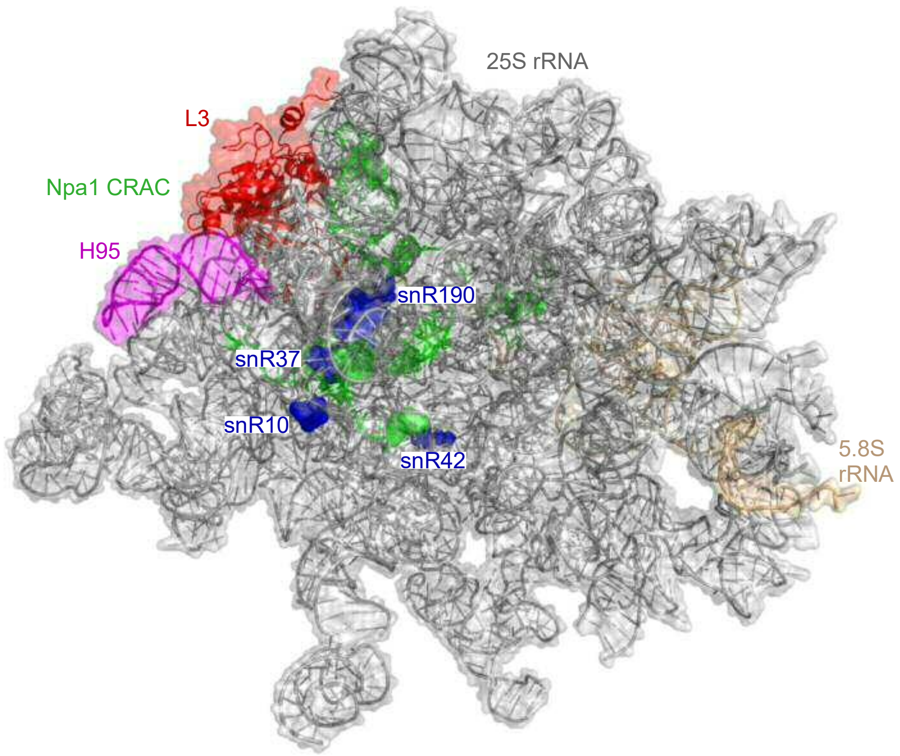

Supplementary Figure 7

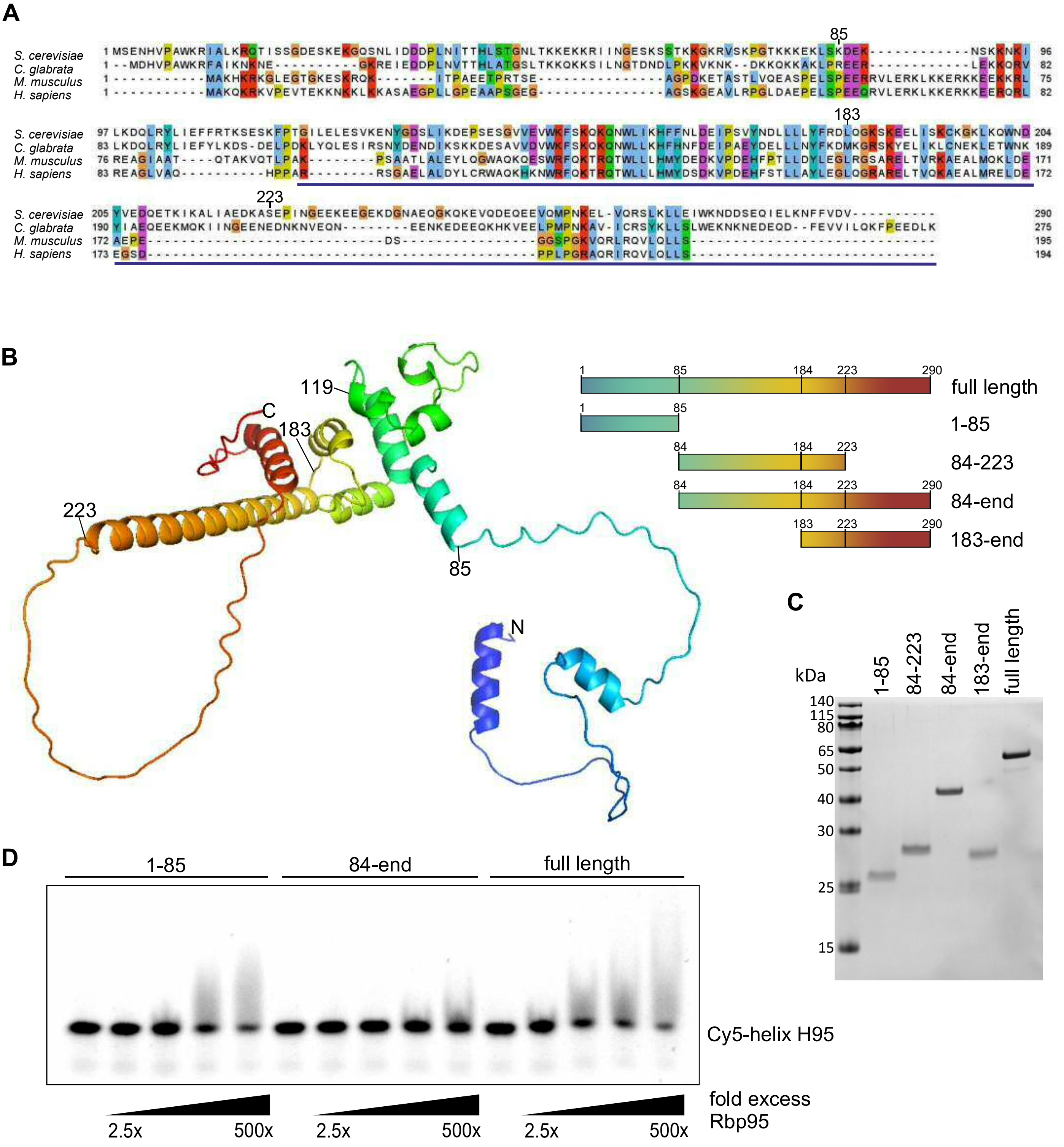

Supplementary Figure 8

A

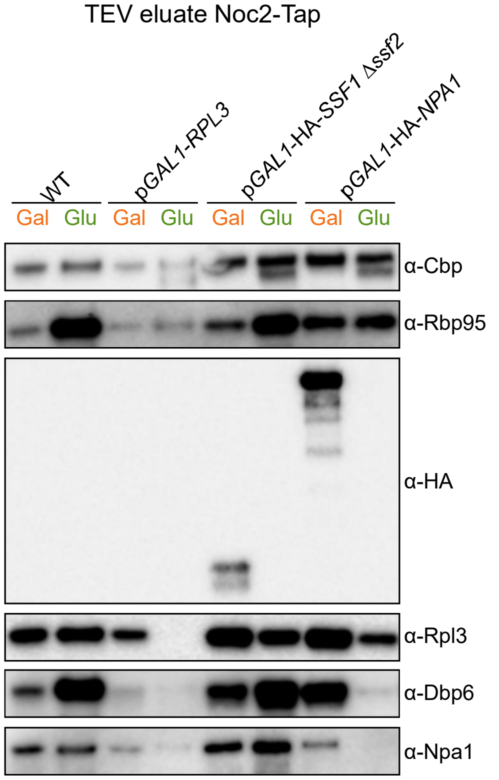

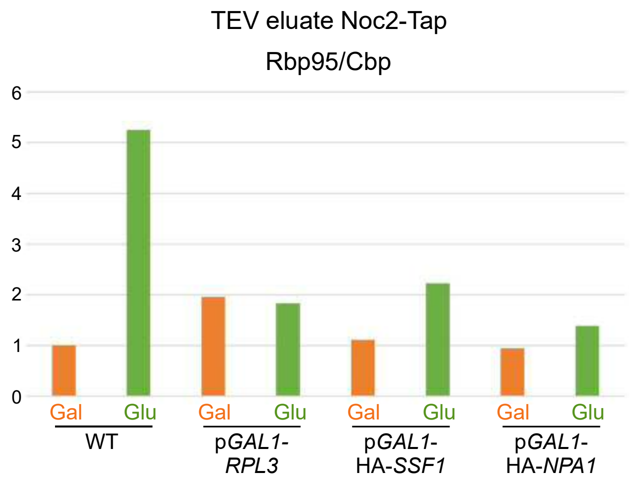

B

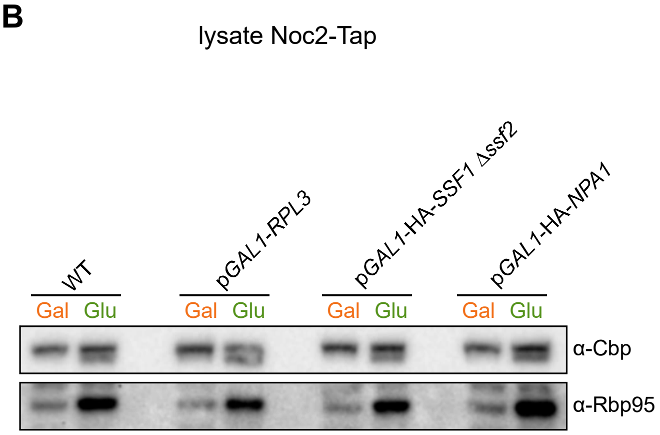

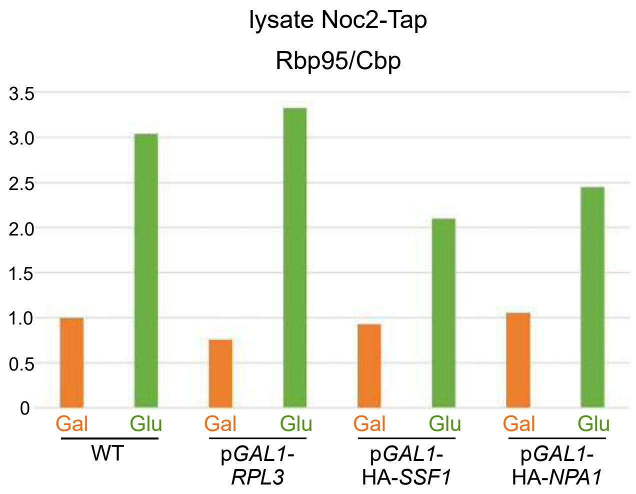

C

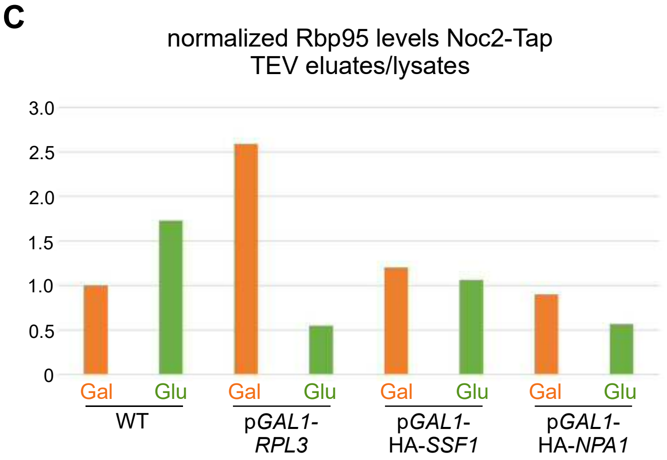

Supplementary Data

Rbp95 binds to 25S rRNA helix H95 and cooperates with the Npa1 complex during early pre-60S particle maturation

Priya Bhutada[1], Sébastien Favre[2], Mariam Jaafar[3,4], Jutta Hafner[1,5], Laura Liesinger[5,6], Stefan Unterweger[1], Karin Bischof[1], Barbara Darnhofer[5,6], Devanarayanan Siva Sankar[2], Gerald Rechberger[1,5], Raghida Abou Merhi[4], Simon Lebaron[3], Ruth Birner-Gruenberger[5,6,7], Dieter Kressler[2], Anthony K. Henras[3], Brigitte Pertschy[1,5]

[1] Institute of Molecular Biosciences, University of Graz, Humboldtstrasse 50, 8010 Graz, Austria.

[2] Unit of Biochemistry, Department of Biology, University of Fribourg, Chemin du Musée 10, 1700 Fribourg, Switzerland.

[3] Molecular, Cellular and Developmental Biology Unit (MCD), Centre de Biologie Intégrative (CBI), Université de Toulouse, CNRS, UPS, 31062, Toulouse, France.

[4] Genomic Stability and Biotherapy (GSBT) Laboratory, Faculty of Sciences, Rafik Hariri Campus, Lebanese University, Beirut, Lebanon.

[5] BioTechMed-Graz, Graz, Austria.

[6] Diagnostic and Research Institute of Pathology, Medical University of Graz, 8010 Graz, Austria

[7] Institute of Chemical Technologies and Analytics, Technische Universität Wien, Getreidemarkt 9/E164, 1060 Vienna, Austria.

Supplementary Figure Legends

Supplementary Figure S1: Ycr016w/Rbp95 is co-purified with G-patch proteins Pfa1 and Pxr1. Final eluates from Pfa1- or Pxr1-TAP purification were analyzed by SDS-PAGE and Coomassie blue staining. The indicated bands, corresponding to Prp43 and Ycr016w/Rbp95, common to both purifications, were identified by mass spectrometry. Bait proteins are indicated by asterisks.

Supplementary Figure S2: *RBP95* deletion does not cause any apparent growth defects. Wild-type and Δ*rbp95* cells were spotted in 10-fold serial dilutions onto SDC and YPD plates, which were incubated for the indicated times at the indicated temperatures.

Supplementary Figure S3: Genetic interactions of *RBP95* with *RPL3*, *DBP6*, and *DBP9*. (**A-C**) *RPL3* (A), *DBP6* (B), and *DBP9* (C) shuffle strains, either harboring wild-type *RBP95* or the Δ*rbp95* deletion at the chromosomal locus, were transformed with plasmids containing either wild-type *RPL3*, *DBP6*, and *DBP9* or the indicated *rpl3*, *dbp6*, and *dbp9* mutant alleles. Transformants were restreaked on SDC-Leu and cells were then spotted in 10-fold serial dilutions onto SD-Leu and 5-FOA-containing plates, which were incubated at 30 ºC for 2 and 5 days, respectively. While the *rpl3-101*, *rpl3-102, dbp6-4*, and *dbp9-5* alleles exhibited synthetic lethality in combination with the Δ*rbp95* null allele*,* the other tested double mutant combinations were viable and their growth phenotypes were further tested in Figure 4A.

Supplementary Figure S4: Quantifications of northern blots shown in Figure 4. **A.** 35S, 27SA$_2$, and 27SA+B signals from the experiment in Figure 4B were quantified and normalized by the signal of 18S rRNA. Signals are indicated relative to the respective wildtype signal. Effects of Δ*rbp95* in combination with *dbp9* or *dbp6* mutations are shown in the left and right charts, respectively. **B.** 35S, 27SA$_2$, and 27SA+B signals from the experiment in Figure 4C were quantified and normalized by the signal of 18S rRNA. Effects of rapid depletion of Rbp95 via the AID-tag upon the indicated times of auxin treatment in the *DBP9* wild-type strain or the *dbp9-1* mutant are shown in the left and right charts, respectively. Signals were calculated relative to the time-point zero (before addition of auxin).

Supplementary Figure S5: 25S rRNA secondary structure (1) with the Rbp95-, Npa1-, and Rpl3-binding sites, as well as the snoRNA base-pairing sites relevant to this study, indicated. The secondary structure was obtained from http://apollo.chemistry.gatech.edu/RiboVision/#SC_LSU_3D. For more details, see legend to Figure 5C.

Supplementary Figure S6: State E pre-60S particle structure (PDB 6ELZ; (2)). For simplicity, all proteins except Rpl3 (red) have been omitted. The 25S rRNA is colored in gray and the 5.8S rRNA in wheat. Helix H95, the binding site of Rbp95, and the Npa1-binding sites are indicated in magenta and

green, respectively. In addition, the residues whose modification is guided by snR37, snR10, and snR42 as well as the hybridization site of snR190 are indicated in blue.

Supplementary Figure S7: Rbp95 binding to RNA. (**A**) Alignment of Rbp95 from *Saccharomyces cerevisiae* with its putative orthologs from *Candida glabrata*, *Mus musculus*, and *Homo sapiens*. The WKF/DUF2373 domain is indicated by a blue bar. (**B**) AlphaFold 2 structural prediction of Rbp95 (3,4) and schematic overview of Rbp95 fragments used for EMSA assays. (**C**) Coomassie blue-stained SDS-PAGE of purified Rbp95 truncated fragments. (**D**) EMSAs were performed as in Figure 6 using the indicated Rbp95 fragments.

Supplementary Figure S8: Rpl3 and Npa1 are required for Rbp95 binding to pre-60S particles. (**A**) Western blot analysis of pre-60S particles purified via Noc2-TAP. *RPL3*, *SSF1*, or *NPA1* were under the control of the glucose repressible *GAL1* promotor. Cells were grown either in galactose-containing (Gal) or glucose-containing (Glu) medium for 18 hours. The right panel indicates quantified Rbp95 levels. To adjust for unequal loading, signals were normalized by the signal of bait protein (Cbp signal). (**B**) Western blot analysis of cell lysates corresponding to the TAP-purification in (A). Quantified Rbp95 levels displayed on the right were normalized by the Cbp signals. (**C**) Relative values of (Cbp-normalized) Rbp95 levels in TEV eluates (A) versus lysates (B).

Supplementary Tables

Supplementary Table 1. Yeast strains

name	genotype	source
W303	*ade2 leu2 his3 trp1 ura3*	(5)
C303	*leu2 his3 trp1 ura3*	(6)
Rbp95-GFP Nop58-mCherry	W303 *MAT*a *NOP58*-yEmCherry::natNT2 *ade3*::kanMX *RBP95*-GFP::*HIS3*MX	this study
Rbp95-TAP	W303 *MAT*a *RBP95*-TAP::*HIS3*MX	this study
DS1-2b	*leu2 his3 trp1 ura2*	(7)
Noc2-TAP	Ds1-2b *MAT*α *NOC2*-TAP::*HIS3*MX	this study
Noc2-TAP Δ*rbp95*	Ds1-2b *MAT*α *NOC2*-TAP::*HIS3*MX *rbp95*::natNT2	this study
Ssf1-TAP	Ds1-2b *MAT*α *SSF1*-TAP::*TRP1*MX	(8)
Ssf1-TAP Δ*rbp95*	Ds1-2b *MAT*α *SSF1*-TAP::*TRP1*MX *rbp95*::natNT2	this study
Nsa1-TAP	Ds1-2b *MAT*α *NSA1*-TAP::*TRP1*MX	(8)
Rix1-TAP	Ds1-2b *MAT*α *RIX1*-TAP::*TRP1*MX	(7)
Arx1-TAP	Ds1-2b *MAT*α *ARX1*-TAP::*TRP1*MX	(7)
Lsg1-TAP	Ds1-2b *MAT*α *LSG1*-TAP::*TRP1*MX	(7)
Prp43-TAP	*MAT*a *leu2 met15 ura3 PRP43*-TAP::*HIS3*MX	Open biosystems
Δ*rbp95*	W303 *MAT*a *rbp95::*kanMX	this study
Δ*rbp95* SL screening strain	W303 *MAT*a *ade3*::kanMX *rbp95*::natNT2 [pHT4467ΔCEN – *RBP95*]	this study
DBP6 shuffle	W303 MATa *dbp6*::kanMX [pRS416-*DBP6*]	(9)
DBP6 shuffle Δ*rbp95*	W303 MATa *dbp6*::kanMX [pRS416-*DBP6*] *rbp95*::hphNT1	this study
DBP9 shuffle	W303 *MAT*a *dbp9*::*HIS3*MX [YCplac33-*DBP9*]	(10)
DBP9 shuffle Δ*rbp95*	W303 *MAT*a *dbp9*::*HIS3*MX [YCplac33-*DBP9*] *rbp95*::hphNT1	this study
RPL3 shuffle	W303 *MAT*a *rpl3*::*HIS3*MX6 [YCPlac33-*RPL3*]	(11)
RPL3 shuffle Δ*rbp95*	W303 *MAT*a *rpl3*::*HIS3*MX6 [YCPlac33-*RPL3*] *rbp95*::hphNT1	this study
DBP9 shuffle *RBP95*-AID	W303 *MAT*a *dbp9*::*HIS3*MX [YCplac33-*DBP9*] *RBP95*-AID::*HIS3*MX *TIR1*-9xmyc::*TRP1*	this study
Noc2-TAP *GAL1*-HA-*SSF1* Δ*ssf2*	Ds1-2b *MAT*α *NOC2*-TAP::*HIS3*MX natNT2::p*GAL1*-3xHA-*SSF1 ssf2*::kanMX	This study
Noc2-TAP *GAL1*-HA-*NPA1*	Ds1-2b *MAT*α *NOC2*-TAP::*HIS3*MX natNT2::p*GAL1*-3xHA-NPA1	This study
Noc2-TAP *GAL1-RPL3*	BY4742 *his3 leu2 ura3 rpl3*::kanMX4 *NOC2*-TAP::*URA3* [p*GAL1-RPL3* (*TRP1*)]	(12)
BY4741	*MAT*a *his3 leu2 met15 ura3*	Euroscarf
Rbp95-HTP (for CRAC)	BY4741 *RBP95*-HTP*::URA3*MX	this study
YDK11-5A WT (for TurboID)	W303 *MAT*α *ade3*::kanMX4	(9)

Supplementary Table 2. *S.cerevisiae* and *E.coli* plasmids.

**name**	**relevant information**	**source**
pRS315 (empty vector)	*CEN*, *LEU2*	(13)
pHT4467ΔCEN – *RBP95*	*cenΔ, ADE3, URA3, RBP95*	this study
pRS315-*RBP95*	*CEN*, *LEU2*, *RBP95*	this study
YCplac111-*NPA1*	*CEN*, *LEU2*, *NPA1*	(14)
YCplac111-*NPA2*	*CEN*, *LEU2*, *NPA2*	(15)
YCplac111-*DBP7*	*CEN*, *LEU2*, *DBP7*	(16)
YCplac111-HA-*RSA1*	*CEN*, *LEU2*, HA-*RSA1*	(9)
YCplac111-*NOP8*	*CEN*, *LEU2, NOP8*	(11)
pHAC111-*RSA3*	*CEN*, *LEU2*, *RSA3*-HA	(11)
pRS416-*DBP6*	*CEN*, *URA3*, *DBP6*	(17)
pRS415-*DBP6*	*CEN*, *LEU2*, *DBP6*	(17)
pRS415-*dbp6-2*	*CEN*, *LEU2*, *dbp6-2*	(9)
pRS415-*dbp6-3*	*CEN*, *LEU2*, *dbp6-2*	(9)
pRS415-*dbp6-4*	*CEN*, *LEU2*, *dbp6-2*	(9)
YCplac33-*DBP9*	*CEN*, *URA3*, *DBP9*	(10)
YCplac111-HA-*DBP9*	*CEN*, *LEU2*, HA-*DBP9*	(10)
YCplac111-HA-*dbp9-1*	*CEN*, *LEU2*, HA-*dbp9-1*	(10)
YCplac111-HA-*dbp9-3*	*CEN*, *LEU2*, HA-*dbp9-3*	(10)
YCplac111-HA-*dbp9-5*	*CEN*, *LEU2*, HA-*dbp9-5*	(10)
YCplac33-*RPL3*	*CEN*, *URA3*, *RPL3*	(11)
YCplac111-*RPL3*	*CEN*, *LEU2*, *RPL3*	(11)
YCplac111-*rpl3-101*	*CEN*, *LEU2*, *rpl3-101*	(11)
YCplac111-*rpl3-102*	*CEN*, *LEU2*, *rpl3-102*	(11)
pCUP111-SV40NLS-yEGFP-(GA)5-TurboID-2xHA (pDK9296)	*CEN, LEU2,* p*CUP1, SV40NLS-yEGFP*-TurboID-2xHA	this study
pCUP111-*RBP95*-(GA)5-TurboID-2xHA (pDK9281)	*CEN, LEU2,* p*CUP1, RBP95*-TurboID-2xHA	this study
pCUP111-*NPA1*-(GA)5-TurboID-2xHA (pDK9282)	*CEN, LEU2,* p*CUP1, NPA1*-TurboID-2xHA	this study
pCUP111-*RPL3*-(GA)5-TurboID-2xHA (pDK9173)	*CEN, LEU2,* p*CUP1, RPL3*-TurboID-2xHA	this study
pET-Duet-His6-Rbp95	Amp^r, T7 promoter/*lac* operator; Rbp95 in MCS1	this study
pET-Duet-His6-Rbp95-1-85	Sequence for Rbp95 1-85 in MCS1	this study
pET-Duet-His6-Rbp95-84-223	Sequence for Rbp95 84-223 in MCS1	this study
pET-Duet-His6-Rbp95-84-end	Sequence for Rbp95 84-end in MCS1	this study
pET-Duet-His6-Rbp95-183-end	Sequence for Rbp95 183-end in MCS1	this study
pFA6a-*HIS3*MX4	for chromosomal deletion	(18)
pFA6a-kanMX4	for chromosomal deletion	(18)
pFA6a-natNT2	for chromosomal deletion	(19)
pFA6a-hphNT1	for chromosomal deletion	(19)
pFA6a GFP::*HIS3*MX	for C-terminal tagging	(18)
pFA6a TAP::*HIS3*MX	for C-terminal tagging	(20)
pBS1539 HTP::*URA3*MX	for C-terminal tagging	(21)

p*CUP1*, *CUP1* promotor; for all other yeast plasmids, the authentic promoters of the respective genes were used.

Supplementary Table 3. Amino acid exchanges of SL mutants.

Mutant #	Complemented by	mutated gene	Amino acid exchanges in protein
4509	*NPA1*	*NPA1*	G1729>R, I1730>N, stop
4512	*NPA2*	*NPA2*	E335>K, I338>F
4510	*RPL3*	*RPL3*	Y283>H
4627	*RPL3*	*RPL3*	Y283>H, (+ silent mutation V162)
4511	*RSA3*	*RSA3*	S34>stop
5009	*RSA3*	*RSA3*	S110>F, insertion with frameshift from 111, stop at 145
4514	*DBP6*	*DBP6*	L346>F
4506	*DBP6, DBP9*	*DBP6*	F580>V
4629	*DBP9*	*DBP9*	P545>T

Supplementary References

1. Petrov, A.S., Bernier, C.R., Gulen, B., Waterbury, C.C., Hershkovits, E., Hsiao, C., Harvey, S.C., Hud, N.V., Fox, G.E. and Wartell, R.M. *et al.* (2014) Secondary structures of rRNAs from all three domains of life, *PloS One,* **9,** e88222.

2. Kater, L., Thoms, M., Barrio-Garcia, C., Cheng, J., Ismail, S., Ahmed, Y.L., Bange, G., Kressler, D., Berninghausen, O. and Sinning, I. *et al.* (2017) Visualizing the Assembly Pathway of Nucleolar Pre-60S Ribosomes, *Cell,* **171,** 1599-1610.e14.

3. Jumper, J., Evans, R., Pritzel, A., Green, T., Figurnov, M., Ronneberger, O., Tunyasuvunakool, K., Bates, R., Žídek, A. and Potapenko, A. *et al.* (2021) Highly accurate protein structure prediction with AlphaFold, *Nature,* **596,** 583–589.

4. Varadi, M., Anyango, S., Deshpande, M., Nair, S., Natassia, C., Yordanova, G., Yuan, D., Stroe, O., Wood, G. and Laydon, A. *et al.* (2022) AlphaFold Protein Structure Database: massively expanding the structural coverage of protein-sequence space with high-accuracy models, *Nucleic Acids Res.,* **50,** D439-D444.

5. Thomas, B.J. and Rothstein, R. (1989) Elevated recombination rates in transcriptionally active DNA, *Cell,* **56,** 619–630.

6. Mitterer, V., Murat, G., Réty, S., Blaud, M., Delbos, L., Stanborough, T., Bergler, H., Leulliot, N., Kressler, D. and Pertschy, B. (2016) Sequential domain assembly of ribosomal protein S3 drives 40S subunit maturation, *Nat Commun.,* **7,** 10336.

7. Nissan, T.A., Bassler, J., Petfalski, E., Tollervey, D. and Hurt, E. (2002) 60S pre-ribosome formation viewed from assembly in the nucleolus until export to the cytoplasm, *EMBO J.,* **21,** 5539–5547.

8. Kressler, D., Roser, D., Pertschy, B. and Hurt, E. (2008) The AAA ATPase Rix7 powers progression of ribosome biogenesis by stripping Nsa1 from pre-60S particles, *J Cell Biol.,* **181,** 935–944.

9. Kressler, D., Doère, M., Rojo, M. and Linder, P. (1999) Synthetic lethality with conditional *dbp6* alleles identifies Rsa1p, a nucleoplasmic protein involved in the assembly of 60S ribosomal subunits, *Mol Cell Biol.,* **19,** 8633–8645.

10. Daugeron, M.C., Kressler, D. and Linder, P. (2001) Dbp9p, a putative ATP-dependent RNA helicase involved in 60S-ribosomal-subunit biogenesis, functionally interacts with Dbp6p, *RNA (New York, N.Y.),* **7,** 1317–1334.

11. de la Cruz, J., Lacombe, T., Deloche, O., Linder, P. and Kressler, D. (2004) The putative RNA helicase Dbp6p functionally interacts with Rpl3p, Nop8p and the novel trans-acting Factor Rsa3p during biogenesis of 60S ribosomal subunits in Saccharomyces cerevisiae, *Genetics,* **166,** 1687–1699.

12. Ohmayer, U., Gil-Hernández, Á., Sauert, M., Martín-Marcos, P., Tamame, M., Tschochner, H., Griesenbeck, J. and Milkereit, P. (2015) Studies on the Coordination of Ribosomal Protein Assembly Events Involved in Processing and Stabilization of Yeast Early Large Ribosomal Subunit Precursors, *PloS One,* **10,** e0143768. First published on Dec 7, 2015.

13. Sikorski, R.S. and Hieter, P. (1989) A system of shuttle vectors and yeast host strains designed for efficient manipulation of DNA in Saccharomyces cerevisiae, *Genetics,* **122,** 19–27.

14. Rosado, I.V. and de la Cruz, J. (2004) Npa1p is an essential trans-acting factor required for an early step in the assembly of 60S ribosomal subunits in Saccharomyces cerevisiae, *RNA (New York, N.Y.),* **10,** 1073–1083.

15. Rosado, I.V., Dez, C., Lebaron, S., Caizergues-Ferrer, M., Henry, Y. and de la Cruz, J. de (2007) Characterization of Saccharomyces cerevisiae Npa2p (Urb2p) reveals a low-molecular-mass complex containing Dbp6p, Npa1p (Urb1p), Nop8p, and Rsa3p involved in early steps of 60S ribosomal subunit biogenesis, *Mol Cell Biol.,* **27,** 1207–1221.

16. Daugeron, M.C. and Linder, P. (1998) Dbp7p, a putative ATP-dependent RNA helicase from Saccharomyces cerevisiae, is required for 60S ribosomal subunit assembly, *RNA (New York, N.Y.),* **4,** 566–581.

17. Kressler, D., de la Cruz, J., Rojo, M. and Linder, P. (1998) Dbp6p is an essential putative ATP-dependent RNA helicase required for 60S-ribosomal-subunit assembly in Saccharomyces cerevisiae, *Mol Cell Biol.,* **18,** 1855–1865.

18. Wach, A., Brachat, A., Alberti-Segui, C., Rebischung, C. and Philippsen, P. (1997) Heterologous HIS3 marker and GFP reporter modules for PCR-targeting in Saccharomyces cerevisiae, *Yeast,* **13,** 1065–1075.

19. Janke, C., Magiera, M.M., Rathfelder, N., Taxis, C., Reber, S., Maekawa, H., Moreno-Borchart, A., Doenges, G., Schwob, E. and Schiebel, E. *et al.* (2004) A versatile toolbox for PCR-based tagging of yeast genes: new fluorescent proteins, more markers and promoter substitution cassettes, *Yeast,* **21,** 947–962.

20. Pausch, P., Singh, U., Ahmed, Y.L., Pillet, B., Murat, G., Altegoer, F., Stier, G., Thoms, M., Hurt, E. and Sinning, I. *et al.* (2015) Co-translational capturing of nascent ribosomal proteins by their dedicated chaperones, *Nat Commun.,* **6,** 7494.

21. Granneman, S., Kudla, G., Petfalski, E. and Tollervey, D. (2009) Identification of protein binding sites on U3 snoRNA and pre-rRNA by UV cross-linking and high-throughput analysis of cDNAs, *Proceedings of the National Academy of Sciences of the United States of America,* **106,** 9613–9618.
