# Supplementary material for: Rbp95 binds to 25S rRNA helix H95 and cooperates with the Npa1 complex during early pre-60S particle maturation
Source: Nucleic Acids Res. 2022 Aug 26;50(17):10053–77. doi: 10.1093/nar/gkac724 (PMC9508819; doi:10.1093/nar/gkac724)
Supplement: gkac724_Supplemental_Files [file gkac724_supplemental_files.zip › LegendsTablesS4S5S6.docx]

**Legends to Supplementary Tables S4, S5 and S6**

**Supplementary Table S4**: Label-free semi-quantitative mass spectrometry results of the experiments displayed in Figure 2D, Figure 8A and Figure 8B. LFQ (label-free quantification) intensities are provided in percent relative to the total intensity of proteins detected in the respective purification.

**Supplementary Table S5**: snoRNAs identified in the CRAC analysis presented in Figure 5. Numbers indicate the respective snoRNA hits per 100,000 reads. Additionally, mutations that occurred in the sequence reads, likely corresponding to the actual crosslinking sites, are indicated.

**Supplementary Table S6:** TurboID mass spectrometry results from the experiment presented in Figure 7. iBAQ (intensity-based absolute quantification) values are indicated.
